# TUT4/7-mediated uridylation of a coronavirus subgenomic RNAs delays viral replication

**DOI:** 10.1038/s42003-023-04814-1

**Published:** 2023-04-21

**Authors:** Ankit Gupta, Yin Li, Shih-Heng Chen, Brian N. Papas, Negin P. Martin, Marcos Morgan

**Affiliations:** 1 Reproductive and Developmental Biology Laboratory, National Institute of Environmental Health Sciences, National Institutes of Health, Durham, NC 27709 USA; 2 Viral Vector Core Facility, National Institute of Environmental Health Sciences, National Institutes of Health, Durham, NC 27709 USA; 3 Integrative Bioinformatics, Biostatistics and Computational Biology Branch, National Institute of Environmental Health Sciences, National Institutes of Health, Durham, NC 27709 USA

**Keywords:** RNA modification, Viral host response, Innate immunity

## Abstract

Coronaviruses are positive-strand RNA viruses with 3′ polyadenylated genomes and subgenomic transcripts. The lengths of the viral poly(A) tails change during infection by mechanisms that remain poorly understood. Here, we use a splint-ligation method to measure the poly(A) tail length and poly(A) terminal uridylation and guanylation of the mouse hepatitis virus (MHV) RNAs. Upon infection of 17-CL1 cells with MHV, a member of the Betacoronavirus genus, we observe two populations of terminally uridylated viral transcripts, one with poly(A) tails ~44 nucleotides long and the other with poly(A) tails shorter than ~22 nucleotides. The mammalian terminal uridylyl-transferase 4 (TUT4) and terminal uridylyl-transferase 7 (TUT7), referred to as TUT4/7, add non-templated uracils to the 3′-end of endogenous transcripts with poly(A) tails shorter than ~30 nucleotides to trigger transcript decay. Here we find that depletion of the host TUT4/7 results in an increased replication capacity of the MHV virus. At late stages of infection, the population of uridylated subgenomic RNAs with tails shorter than ~22 nucleotides is reduced in the absence of TUT4/7 while the viral RNA load increases. Our findings indicate that TUT4/7 uridylation marks the MHV subgenomic RNAs for decay and delays viral replication.

## Abstract (web summary)

The mammalian terminal uridylyl-transferases TUT4 and TUT7 uridylate the mouse hepatitis virus subgenomic RNAs with poly(A) tails shorter than 22 nucleotides to promote their decay and therefore delay viral replication.

## Introduction

Coronaviruses have single-stranded RNA genomes which are used to generate subgenomic transcripts. Both the viral genome and the subgenomic RNAs (sgRNAs) are 3′ polyadenylated^1^. During the course of the infection, the poly(A) tails change in length which is believed to contribute to viral replication. Typically, at 8 hours post-infection (hpi), the viral RNA (vRNA) poly(A) tails are ~60 nucleotides long and gradually decrease in size as the infection progresses. By 48 hpi, the poly(A) tails shorten to less than ~40 nucleotides^2,3^. However, little is known about the mechanisms regulating the vRNA poly(A) tail size.

Once the virus infects the cells, the genomic RNA (gRNA) is translated into various proteins required for viral RNA processing, such as the viral RNA-dependent RNA polymerase nps12^1^. In addition to replicating the gRNA, nsp12 also synthesizes each complementary sgRNA starting from the 3′-end of the gRNA. In this way, the gRNA and all sgRNAs share the same 3′ UTR. While both the gRNA and the sgRNAs are translated, only the gRNA is incorporated into the viral particles. The contributions of vRNA 3′-end processing to the differential treatment of the genomic and subgenomic RNAs remains poorly understood.

Recent studies have shown that host terminal nucleotidyl-transferases (TENTs) can add non-templated nucleotides to the poly(A) tails of different viruses to affect their replication capacity^4^. Two mammalian TENTs, terminal uridylyl-transferase 4 (TUT4) and terminal uridylyl-transferase 7 (TUT7), hereafter referred to as TUT4/7 transfer U residues to the 3′-end of the influenza A virus mRNAs, but not to its genomic RNAs nor complementary RNAs^5^. The terminal uridylation of the influenza A mRNAs delays the replication of the virus, presumably by targeting the mRNAs for degradation. TUT4/7 are redundant proteins required for normal development in mammals; their depletion leads to aberrant cell differentiation and perinatal death^6–8^. In the cytoplasm, TUT4/7 uridylate transcripts with poly(A) tails shorter than 30 nucleotides, which are no longer bound and protected by poly(A) binding proteins (PABP)^9^. Terminal uridylation also plays a role in the innate immune response against viruses in *C. elegance* alongside siRNA targeting pathways^5^.

Viruses can use endogenous RNA-modifying enzymes to facilitate their replication as well. In the case of the hepatitis B virus, the terminal nucleotidyltransferase 4 A (TENT4A) and terminal nucleotidyltransferase 4B (TENT4B) extend the poly(A) tail of the vRNA promoting its stabilization and facilitating viral replication^10–12^. TENT4A and TENT4B are non-canonical poly(A) polymerases that occasionally incorporate G residues while elongating the RNA 3′-end resulting in poly(A) tails with interspersed Gs^13^. The presence of G residues in the tail provides stability to the transcript by inhibiting the activity of deadenylases^11^. TENT4A and TENT4B have also been shown to promote the viral replication of the hepatitis A virus^14^. Thus, endogenous TENTs can also be co-opted by viruses.

In this study, we investigate the role of TENTs in the replication of the mouse hepatitis virus (MHV), a member of the Betacoronavirus genus. To determine whether the poly(A) tails of the viral gRNA and sgRNAs are targets of TUT4/7 or the TENT4s, we used a splint-ligation protocol to capture the 3′-end of the vRNAs. Although we did not detect a high frequency of guanylated tails, the hallmark of TENT4s activity, we found two populations of uridylated vRNAs, one with poly(A) tails shorter than ~22 nucleotides and the other one with poly(A) tails longer than ~44 nucleotides. Uridylation of tails shorter than ~22 nucleotides occurred only on sgRNAs. Depletion of TUT4/7 reduced the levels of uridylation of short tails, increased the vRNA load, and enhanced viral replication. We propose that TUT4/7 directly target sgRNAs to promote their decay, which in turn limits the replication capacity of the virus.

## Results and discussion

### The MHV RNA has two distinct populations of uridylated poly(A) tails

**Fig. 1:**
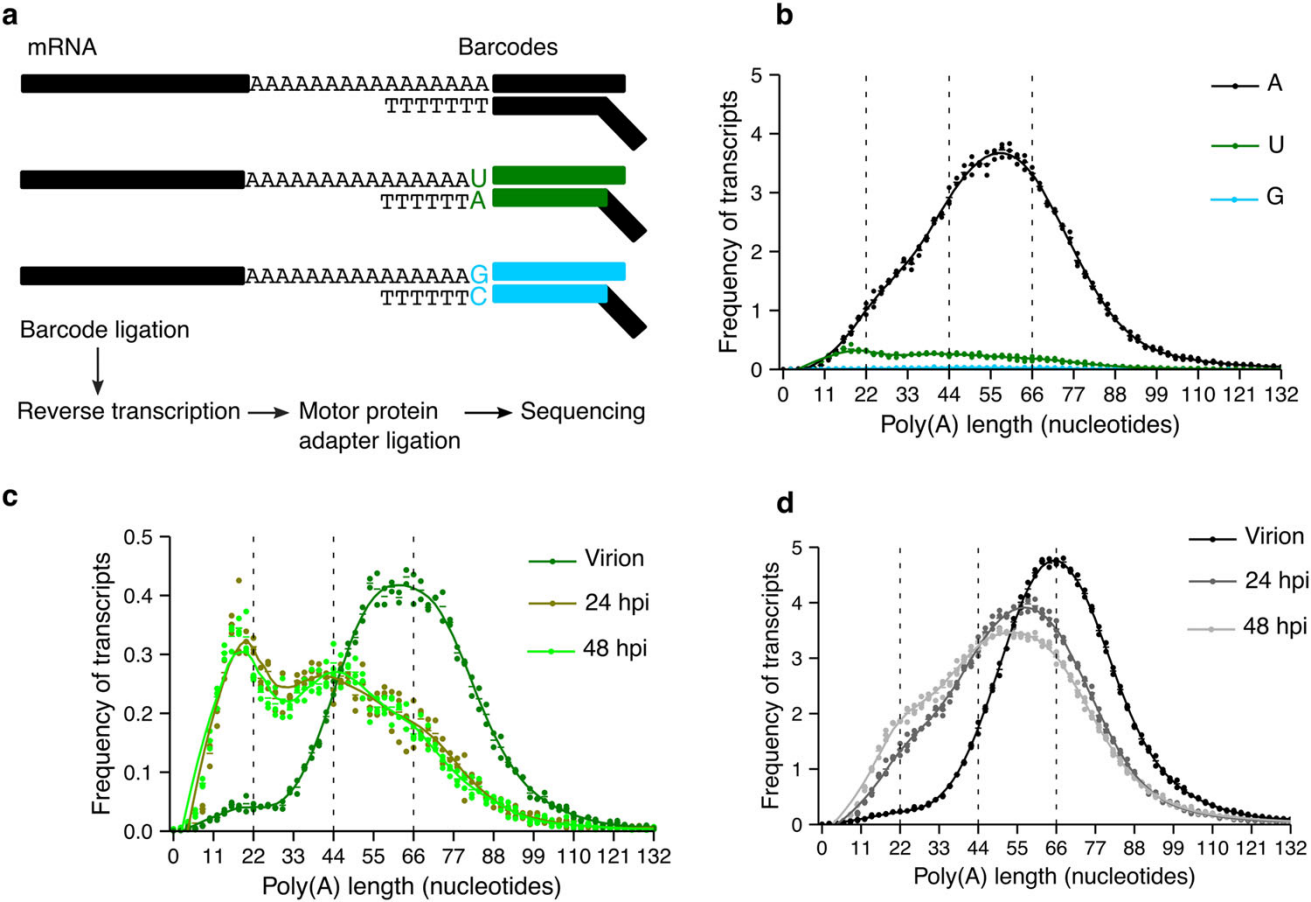
MHV RNA is uridylated during infection. **a** A diagram of the splint-ligation strategy used to capture RNA 3′-end modifications using barcoded oligos is shown. **b** The poly(A) tail length profile of MHV RNA from 17-CL1 infected cells 24 hours post-infection (hpi) is shown in black for tails without terminal modifications, in green for terminally uridylated tails, and in cyan for terminally guanylated tails. **c** The poly(A) tail length profile of terminally uridylated tails of the MHV virion RNA and MHV RNA from 17-CL1 infected cells at 24 and 48 hpi are indicated in dark green, olive, and light green, respectively. Uridylated reads represent 9.9%, 8.8%, and 8.7% of total reads in the virion RNA, the vRNA 24 hpi, and the vRNA 48 hpi, respectively. The profile of the uridylated transcripts at 24 hpi is the same as shown in **b**. **d** The poly(A) tail length profile of the MHV virion RNA and MHV RNA from 17-CL1 cells infected at 24 and 48 hpi are depicted in black, gray, and light gray, respectively. Dots represent the frequency of poly(A) tail length for each sample, and the horizontal bars show the mean frequency of poly(A) tail length per condition (*n* = 2 for the virion and *n* = 3 for 24 and 48 hpi).

We and others have previously shown that TENTs are able to extend the poly(A) tails of vRNAs affecting the replication capacity of the viruses^5,10,12^. To determine whether the TENTs act upon MHV RNAs, we developed a splint-ligation protocol to capture terminal uridylation and terminal guanylation, the signature modifications of TUT4/7 and TENT4s, respectively (Fig. 1a, Supplementary Fig. 1a, b, and Supplementary Tables 1 and 2). The lack of a strong guanylation signature suggests that, at least in our system, the MHV RNA is not being polyadenylated by TENT4s. Still, functional analyses such as TENT4s inhibition or depletion would be required to further evaluate this possibility. However, in infected 17-CL1 cells, we found vRNA uridylation in poly(A) tails ~22 nucleotides long 24 hpi together with a minor peak of uridylation on vRNAs with poly(A) tails ~44 nucleotides long (Fig. 1b, c). A peak of uridylation at tails ~44 nucleotides long was also found in NCTC cells (Supplementary Fig. 1c).

To determine whether the uridylation profile changed during the MHV infection, we examined the virion RNA and the viral RNA at 24 and 48 hpi. While the virion RNA shows uridylation of tails ~55 to ~66 nucleotides long (Fig. 1c), the peak of uridylation shifts to ~22 nucleotides long tails by 24 hpi and remains at ~22 nucleotides 48 hpi (Fig. 1c). The presence of short terminal uridylated poly(A) tails suggests that vRNAs could be targeted by the endogenous uridylyl-transferases TUT4/7, which can transfer single uracils (mono-uridylation) or a few uracils (oligo-uridylation) to the 3′-end of an RNA. Consistently, we observed that MHV vRNAs are both mono- and oligo-uridylation in our system (Supplementary Table 1). To confirm by an independent method that vRNAs were uridylated, we circularized the virion RNA, followed by cloning and Sanger sequencing of amplicons spanning the ligation site. The protocol allowed us to capture in an unbiased manner the terminal nucleotides of the vRNA. We found that MHV RNAs are frequently terminally uridylated but not guanylated as expected (Supplementary Table 3).

The poly(A) profile of the virus also changes during infection (Fig. 1d). The poly(A) tail of the virion peaks at ~66 nucleotides and has a symmetric distribution, while the poly(A) tail of the virus shortens by 24 hpi peaking at ~55 nucleotides with a shoulder at ~22 nucleotides. The shortening of the poly(A) tails continues at 48 hpi as the population of RNAs with poly(A) tails ~22 nucleotides long increases (Fig. 1d). Overall, these results show that during MHV infection of 17-CL1 cells, there is a progressive shortening of the poly(A) tail of the viral RNA that takes place along with an increase in the uridylation of short tails.

### The uridylation of short tails is only observed in sgRNAs

**Fig. 2:**
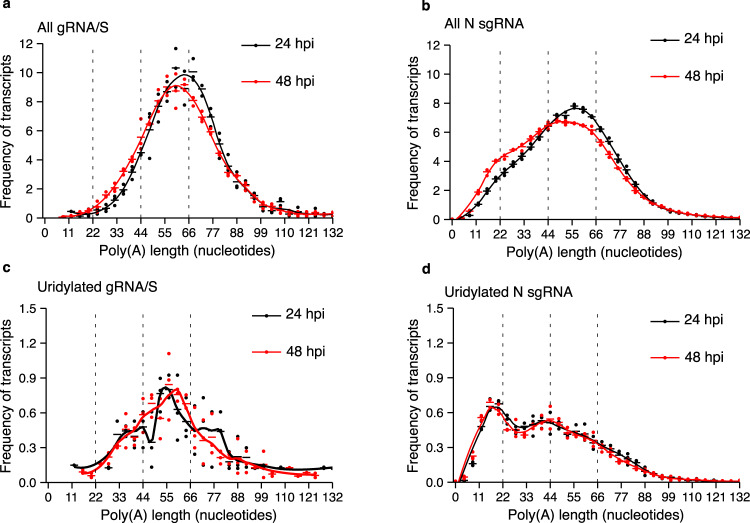
N sgRNAs with poly(A) tails shorter than ~22 nucleotides are uridylated. **a**, **b** The poly(A) tail length profiles of the gRNA/S species (**a**) or the N sgRNA (**b**) from 17-CL1 infected cells at 24 and 48 hours post-infection (hpi) are depicted in black and red, respectively. **c** The uridylation profiles of the gRNA/S species from 17-CL1 infected cells at 24 hpi (black) and 48 hpi (red) are shown. Uridylated reads represent 7.6% and 8.2% of total reads at 24 hpi and 48 hpi, respectively. **d** The uridylation profiles of the N sgRNA from 17-CL1 infected cells at 24 hpi (black) and 48 hpi (red) are shown. Uridylated reads represent 8.8% and 8.6% of total reads at 24 hpi and 48 hpi, respectively. Dots represent the frequency of poly(A) tail length for each sample, and the horizontal bars show the mean frequency of poly(A) tail length per condition (*n* = 3 for all conditions).

We next sought to understand whether the uridylation profile depended on the type of vRNA. In addition to facilitating the determination of transcripts’ poly(A) tail lengths, our long-read library preparation protocol allowed us to simultaneously differentiate between different vRNAs and opened the possibility of examining the poly(A) tail profile for individual vRNA species. However, given that most of our reads did not extend beyond the spike sgRNA (S sgRNA), we were unable to differentiate between the gRNA and the S sgRNA; thus, we analyzed both these populations together, and we refer to them as gRNA/S. The poly(A) profile of the gRNA/S species showed a unimodal distribution throughout the infection, while the nucleocapsid sgRNA (N sgRNA) presented an accumulation of poly(A) tails shorter than ~22 nucleotides over the course of the infection (Fig. 2a, b). Other sgRNAs with sufficient read coverage, such as the membrane protein sgRNA (M sgRNA) and the envelope protein sgRNA (E sgRNA), showed a distribution of poly(A) tail length similar to that observed for the N sgRNA (Supplementary Fig. 2a, b). These results show that the dynamic changes in poly(A) profiles depend on the type of vRNA, with the most abundant sgRNAs (i.e., E, N, and M) showing a shift of the distribution towards shorter tails.

We then examined the uridylation profile of the different vRNAs to determine whether the uridylation profile was also dependent on the type of vRNA. As for the poly(A) profile, we observed that the gRNA/S species presented a unimodal uridylation distribution which remained centered at ~60 nucleotides as the infection progressed (Fig. 2c). The N sgRNA instead showed a distribution of uridylated tails with a peak at ~22 nucleotides at 24 and 48 hpi (Fig. 2d). The same distribution was observed for the other sgRNAs (Supplementary Fig. 2c, d Supplementary Table 4). Taken together, these results show that both the poly(A) tail and terminal uridylation profiles not only change during infection, but they do so in a vRNA species-specific manner, with both the increase in short tails and short tail uridylation occurring on the most abundant sgRNA (i.e., M, N, E), but not in the gRNA/S species.

### TUT4/7-depletion facilitates viral replication and increases the viral load

**Fig. 3:**
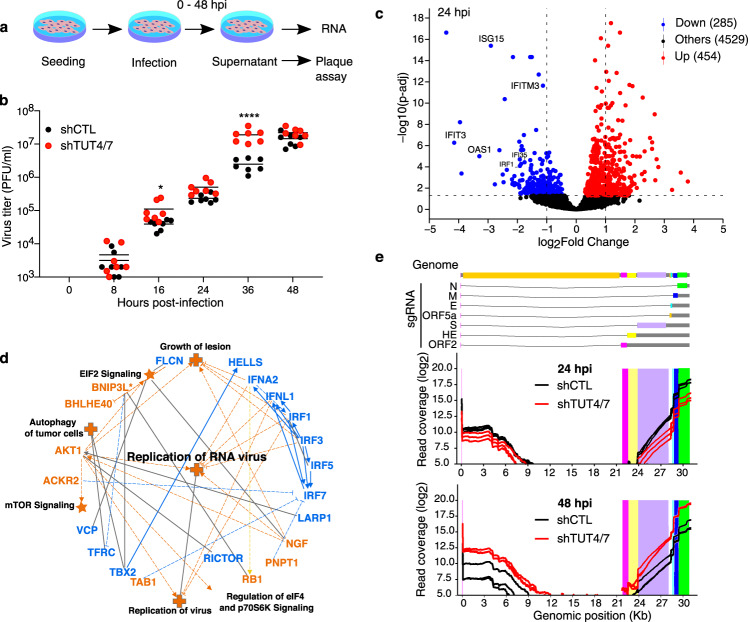
Depletion of TUT4/7 impacts viral RNA metabolism and replication. **a** A schematic representation of the strategy used for viral infection and sample collection is shown. **b** The quantification of viral particles in the supernatant of MHV-infected shCTL and shTUT4/7 cells at different time points, 0, 8, 16, 24, 36, and 48 hours post-infection (hpi), using plaque assay is shown. Virus quantification from shCTL and shTUT4/7 samples are indicated in black and red, respectively. Each dot represents one biological replicate (*n* = 7), and the bars indicate the mean value for each condition at every time point (two-way ANOVA; ****p < 0.0001; *p < 0.05). **c** Volcano plot showing differential gene expression between shCTL and shTUT4/7 cells at 24 hpi (*n* = 3). Significantly upregulated genes are indicated in red, and significantly down-regulated genes in blue. Transcripts with an adjusted *p* value lower than 0.05 were considered significant. Genes involved in the interferon signaling pathway are labeled. **d** Graphical representation of Ingenuity Pathway Analysis (IPA) results comparing the transcriptome of control and TUT4/7-depleted cells at 24 hpi. Upregulated pathways are shown in orange, and down-regulated pathways are in blue. Disease pathways are indicated with crosses, and canonical pathways are indicated with stars. Full lines and dotted lines represent direct and indirect interactions, respectively. **e** A schematic representation of the MHV genomic and subgenomic RNAs (sgRNA) is shown in the upper panel. The normalized viral read coverage profile across the viral genome at 24 hpi (middle panel) and 48 hpi (lower panel) are shown. Read coverage profiles of MHV-infected shCTL and shTUT4/7 cells are indicated in black and red, respectively.

TUT4/7 are known to promote mRNA decay by uridylating poly(A) tails shorter than 30 nucleotides^9^. Thus, we speculated that TUT4/7 could be uridylating the vRNAs with poly(A) tails shorter than 22 nucleotides, potentially impacting the replication cycle of MHV. To determine whether TUT4/7-depletion affects viral replication, we performed plaque assays to quantify the production of viral particles from 17-CL1 control (shCTL) and TUT4/7-depleted (shTUT4/7) cells at 0, 8, 16, 24, 36, and 48 hpi (Fig. 3a, b and Supplementary Fig. 3a, b). While shCTL and shTUT4/7 cells release a similar number of viral particles at 24 hpi (Fig. 3b), shTUT4/7 cells produce significantly more particles at 36 hpi. By 48 hpi, the particles released by both cell lines reach similar levels again. To better understand the infection dynamics, we repeated the plaque assay experiments at time points closer to 36 hpi. We confirmed that the increase in viral particle production in the absence of TUT4/7 occurs in a time window centered around 36 hpi (Supplementary Fig. 3d). Thus, although TUT4/7 do not prevent the formation of viral particles, they delay viral replication.

TUT4/7 are required for fertility and to modulate innate immune responses^5–7,15^. We hypothesized that depletion of TUT4/7 could facilitate the replication of the virus by altering the cellular response to pathogens, as previously reported^16–19^. To explore this possibility, we performed gene expression analysis of shCTL and shTUT4/7 cells before infection (Supplementary Fig. 3c). Absence of TUT4/7 led to the dysregulation of a subset of ~140 transcripts (Supplementary Fig. 4a). Gene ontology analysis of the upregulated transcripts in shTUT4/7 cells revealed an enrichment for inflammation and response to external stimulus pathways (Supplementary Fig. 4b). Interleukin 33 (IL33), a cytokine expressed by fibroblasts and cells in direct contact with the environment, was upregulated approximately ten-fold in the shTUT4/7 line^20^ (Supplementary Fig. 4a, c). Thus, the increased capacity of the virus to replicate in TUT4/7-depleted cells is not due to a suppressed innate immune state before infection.

To gain insights into the changes in the innate immune response during viral infection in the absence of TUT4/7, we compared the gene expression profile of TUT4/7-depleted cells and controls at 24 hpi, just before we detected differences in viral particle production. We observed a significant upregulation of genes involved in eukaryotic translation initiation factor 2 (EIF2) signaling and a significant downregulation of genes involved in the interferon response (Fig. 3c, d and Supplementary Fig. 4d). Thus, the combined dysregulation of these pathways results in an enhanced replication capacity of the RNA virus (Fig. 3c, d). The upregulation of translation in TUT4/7-depleted cells was also maintained at 48 hpi (Supplementary Fig. 4e, f). Changes to viral proteins, such as viral protein nsp15, a uridylate-specific endoribonuclease, have also been shown to shape the innate immune response of the host cells during infection^21^. Together these results indicate that the enhanced innate immune state of the TUT4/7-deficient cells reverts when the cells are infected with the virus to facilitate viral replication.

Since TUT4/7 are known to promote RNA decay, we sought to determine whether they could affect the viral RNA load during infection. To address this point, we normalized viral RNA reads to the endogenous transcripts and plotted the normalized read coverage across the viral genome (Fig. 3e). The coverage plot showed the characteristic higher accumulation of the shorter sgRNAs compared to the longer ones^22^. While the complementary sgRNAs are being generated, the RNA-dependent RNA polymerase switches templates from a characteristic sequence immediately upstream of the sgRNAs’ open reading frames (ORFs) to the leader sequence at the 5′-end of the virus genome. The template switching results in the staggered decrease in the read coverage immediately upstream of the sgRNAs ORFs. In some instances, the template switching occurs in the vicinity of the leader sequence rather than precisely on the leader sequence itself, which accounts for the increase in 5′ coverage.

When comparing the read coverage between control and mutant cells, we observed that at 24 hpi, there was less vRNA accumulation in the TUT4/7-depleted cells compared to controls, probably due to the activation of the IL33 pathway in the absence of TUT4/7 (Fig. 3e, Supplementary Table 2 and Supplementary Fig. 4a–c). However, by 48 hpi, the situation reverted with more accumulation of viral RNA on the TUT4/7-depleted cells (Fig. 3e, Supplementary Table 2). The reduced level of RNA in the TUT4/7-depleted cells 24 hpi could be attributed to a delay in the infection progression. However, at 48 hpi, when both shCTL and shTUT4/7 cells had produced a similar number of viral particles, the accumulation of vRNA in the TUT4/7-depleted cells could be explained by a reduction in vRNA decay. Indeed, while the vRNA load continues to increase during the infection in the shTUT4/7 mutants, the vRNA load peaks in the shCTL samples at 24 hpi (Supplementary Table 2). Thus, we speculate that the reduction in the vRNA load in control samples could be mediated by TUT4/7.

### Uridylation of short poly(A) tails is dependent on TUT4/7

**Fig. 4:**
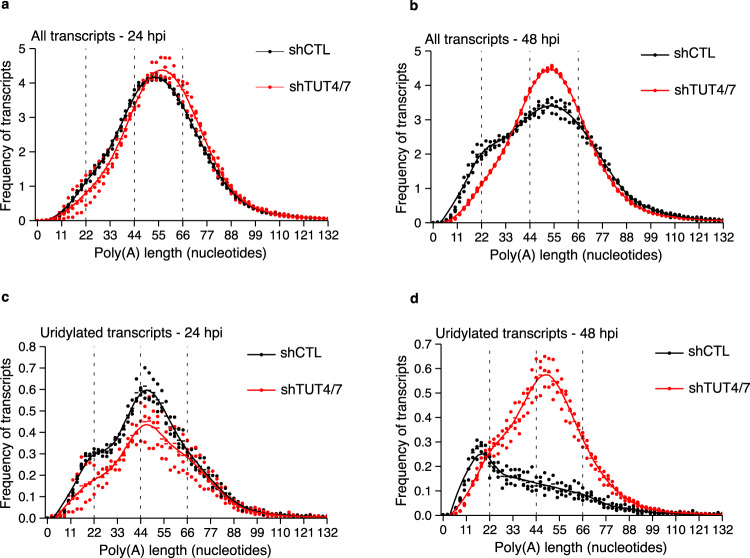
Depletion of TUT4/7 prevents the accumulation of short uridylated tails. **a**, **b** The vRNA poly(A) tail length profiles from shCTL (black) and shTUT4/7 (red) infected cells at 24 hours post-infection (hpi) (**a**) and 48 hpi (**b**) are shown. **c** The poly(A) tail length profiles of terminally uridylated vRNA from shCTL (black) and shTUT4/7 (red) infected cells at 24 hpi. Uridylated reads represent 13.9% and 10.5% of total reads in shCTL and shTUT4/7 cells, respectively. **d** The poly(A) tail length profiles of terminally uridylated MHV RNA from shCTL (black) and shTUT4/7 (red) infected cells at 48 hpi. Uridylated reads represent 5.4% and 12.9% of total reads in shCTL and shTUT4/7 cells, respectively. Dots represent the poly(A) tail length frequency for each sample, and the horizontal bars show the mean frequency of poly(A) tail length per condition (*n* = 3 for each condition).

To gain mechanistic insight into the pathways that regulate the poly(A) tail dynamics of the virus, we monitored changes in 3′-end regulation during MHV infection in 17-CL1 shCTL and shTUT4/7 cells. Although differences between the endogenous shCTL and shTUT4/7 transcriptome were maintained throughout the infection (Supplementary Fig. 3c), these differences were not reflected by changes in the global poly(A) or uridylation profiles of the endogenous transcripts (Supplementary Fig. 5a-f). The limited reduction in uridylation levels of the endogenous transcripts could be explained by initial low levels of uridylation that can still be achieved with residual amounts of TUT4/7 after knockdown (Supplementary Table 5). The vRNAs poly(A) and uridylation profiles instead undergo profound changes between 24 and 48 hpi but only in the presence of TUT4/7. In TUT4/7-depleted cells, no accumulation of viral transcripts with poly(A) tails ~22 nucleotides long was observed at 24 hpi, and the population could still not be detected at 48 hpi (Fig. 4a, b). The uridylation peak at tails shorter than ~22 nucleotides also failed to materialize. On the contrary, the uridylation peak at ~44 nucleotides, already prominent at 24 hpi, consolidated at 48 hpi (Fig. 4c, d). These results suggest that TUT4/7 directly uridylate the viral RNA with poly(A) tails shorter than ~22 nucleotides.

### Reduced uridylation of short tails only alters the poly(A) profile of sgRNAs

**Fig. 5:**
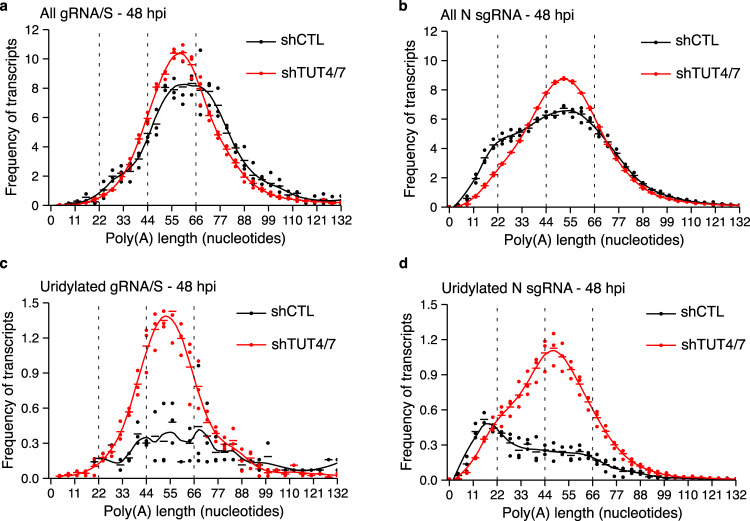
TUT4/7-depletion alters the uridylation profile of the N sgRNA. **a**, **b** The poly(A) tail length profiles of the gRNA/S species (**a**) or the N sgRNA (**b**) from infected shCTL (black) and shTUT4/7 (red) cells at 48 hours post-infection (hpi) are shown. **c** The poly(A) tail length profiles of terminally uridylated gRNA/S species from shCTL (black) and shTUT4/7 (red) infected cells at 48 hpi. Uridylated reads represent 4.7% and 12.8% of total reads in shCTL and shTUT4/7 cells, respectively. **d** The poly(A) tail length profiles of terminally uridylated N sgRNA from shCTL (black) and shTUT4/7 (red) infected cells at 48 hpi. Uridylated reads represent 5.3% and 12.7% of total reads in shCTL and shTUT4/7 cells, respectively. Dots represent the frequency of poly(A) tail length for each sample, and the horizontal bars show the mean frequency of poly(A) tail length per condition (*n* = 3 for each condition).

Next, we sought to examine how the poly(A) and uridylation profiles of the different vRNA species changed upon depletion of TUT4/7. The poly(A) profile of the gRNA/S species, which did not change during infection, also preserved their distribution after TUT4/7-depletion (Fig. 5a). In contrast, the shift toward shorter tails of the N sgRNA was hampered in the absence of TUT4/7 (Fig. 5b). The same lack of poly(A) tail shortening was observed for other sgRNAs upon the depletion of the TUTs (Supplementary Fig. 6a, b).

Finally, we examined whether the uridylation profile of the different vRNA species was affected by the TUTs. We hypothesized that, as for the global distribution, the sgRNAs with uridylated short poly(A) tails would present a shift toward the uridylation of tails ~44 nucleotides long. Indeed, while the uridylation profile of the gRNA/S species was not affected in the shTUT4/7 cells compared to controls (Fig. 5c), the N sgRNA showed a shift of uridylation from short to long tails (Fig. 5d). We observed the same distribution for the other sgRNAs throughout the course of the infection (Supplementary Fig 6c, d, and Supplementary Fig. 7a–h, Supplementary Table 4).

Taken together, these results are consistent with a model in which all vRNA species have poly(A) tails longer than ~55 nucleotides, but only a subset of the most abundant sgRNA have their tails shortened as the infection progresses. These short poly(A) tails are uridylated by TUT4/7, which are known to promote transcript degradation^9^. The accumulation of vRNAs in the absence of TUT4/7 is consistent with the role of these enzymes in mRNA decay. Upon TUTs depletion, these transcripts revert to poly(A) and uridylation profiles similar to those of the gRNA/S species. Thus TUT4/7 is a critical component of the pathways that shape the poly(A) profile of the most abundant sgRNA species, with implications on the replication capacity of the virus.

## Conclusions

The high transmissibility of coronaviruses requires a multilayered approach to minimize infections and symptoms. Identification of endogenous factors that regulate viral RNA processing is critical for developing novel treatments. Here we found that coronaviruses poly(A) tails show two types of uridylation, one of which is dependent on the endogenous proteins TUT4/7. Depletion of these proteins promotes viral replication and increases viral RNA load, revealing an unexplored pathway in the RNA processing of coronaviruses.

## Methods

### Cell lines and tissue culture

The murine 17-CL1 cell line (derived from 3T3 cells, BEI Resources, Cat. No. NR-53719) was maintained in Dulbecco Modified Eagle Medium (DMEM, Life Technologies) supplemented with 10% Fetal Bovine Serum (FBS, Gemini Bio-Products), L-glutamine (Invitrogen), and Sodium pyruvate (Invitrogen). The NCTC clone 1469 cell line (derivative of NCTC 721, ATCC, Cat. No. CCL-9.1) was maintained in DMEM medium with 10% FBS, l-glutamine, and 1× MEM non-essential amino acids (Millipore Sigma). None of the cell lines were authenticated. Cell lines were tested for mycoplasma throughout the course of the experiments. Cells lines that tested negative for mycoplasma were propagated. Cell lines that tested positive were immediately discontinued and replaced with clean ones.

### Production of shRNA lentiviral vectors and generation of 17-CL1 shCTL and shTUT4/7 stable cells

The mouse pGIPZ lentiviral shRNA vectors (ThermoScientific) were provided by the NIEHS Epigenetics Core facility; detailed information can be found on the website, https://horizondiscovery.com/en/gene-modulation/knockdown/shrna/products/gipz-lentiviral-shrna. The shRNA sense-targeting sequences used in this study are summarized in Supplementary Table 6. All lentiviruses were packaged in HEK293T cells according to published protocols^23^. Briefly, cells were transiently transfected with psPAX2, a gift from Didier Trono (Addgene, Cat. No. 12260), pMD2.G, a gift from Didier Trono (Addgene, Cat. No. 12259), and pGIPZ shRNA vectors carrying shCTL, shTUT4 or shTUT7 using Lipofectamine 2000. Supernatants were collected 48 hours post-transfection, aliquoted, and stored at −80 °C. Titers were determined using digital droplet PCR to measure the number of lentiviral particles integrated into the transduced HEK293T genome. To generate 17-CL1 shCTL and shTUT4/7 stable lines, cells were seeded in 6-well plates (20,000 cells per well). The next morning, cells were infected with a multiplicity of infection (MOI) of 20 for shCTL or 10 MOI for shTUT4, and 10 MOI for shTUT7 lentiviruses. The next day, the medium was replaced with 2 µg/ml puromycin-supplemented medium. Cells were maintained for 48 hours under selection to obtain the shCTL and shTUT4/7 stable lines. The reduction of *Tut4* and *Tut7* levels in shTUT4/7 cells was confirmed by qPCR.

### RNA isolation and qPCR analysis

Total RNA was extracted from infected and not infected 17-CL1 shCTL or shTUT4/7 cells using the RNeasy Mini Kit (Qiagen, Cat. No. 74104) or from MHV virion using TRIzol. First-strand cDNA synthesis was performed using SuperScript II reverse transcriptase according to the manufacturer’s protocol (Invitrogen). The expression levels of *Tut4* and *Tut7* were measured by qPCR using SYBR green assays (Applied Biosystems). The qPCR protocol consisted of a denaturation step of 20 seconds at 95 °C followed by 40 cycles of 3 seconds at 95 °C and 30 seconds at 60 °C. The sequences of qPCR primers are shown in Supplementary Table 7. Cycle threshold (Ct) values were obtained using the ABI PRISM 7900 Sequence Detection System and analysis software (Applied Biosystems). Each sample was normalized to mouse *Gapdh* expression, and fold changes were calculated relative to 17-CL1 shCTL cells Ct values.

### MHV infection

The MHV strain S was purchased from ATCC (Cat. No. VR-766). For MHV infection, 17-CL1 cells were seeded into 6-well plates (400,000 cells per well) overnight and infected with 10 MOI MHV for 0, 8, 16, 24, 30, 36, 42, and 48 hours. The supernatants were collected for MHV plaque assay, and cells were harvested for RNA isolation.

### MHV plaque assays

17-CL1 cells were used for the plaque assay with MHV titers expressed as PFUs/ml. Briefly, cell monolayers in 24-well plates (75,000 cells per well) were adsorbed for an hour with a series of 10-fold dilutions of either MHV inoculum or cell culture supernatants. The infected monolayers were left for 3–4 days under 0.8% methylcellulose overlay (in EMEM medium with 2% FBS, 10 mM of HEPES, 2 mM of l-Glutamine, 100 units of penicillin and 0.1 mg of streptomycin) until plaques become visible. The cell monolayers were stained with 2% crystal violet in 10% ethanol for at least an hour. The stained monolayers were washed and air-dried, and clear plaques were counted manually under a light microscope.

### Synthesis of RNA standards

In vitro transcribed RNAs were used as internal poly(A) standards. Barcoded oligos (Supplementary Table 8) were used to generate PCR amplicons from the plasmid pSpCas9(BB)−2A-GFP (PX458), a gift from Feng Zhang (Addgene, Cat. No. 48138)^24^. The amplification reaction contained 1 µl of the Phusion polymerase (NEB, Cat. No. M0530L), 1× Phusion polymerase buffer, 1 pg/µl of the vector, 1 mM dNTP mix and 1 µM of each primer in a total volume of 100 µl. The amplification reaction consisted of an initial denaturation at 95 °C for 3 minutes followed by 34 cycles of 30 seconds at 95 °C, 30 seconds at 58 °C, and 2 minutes at 72 °C. The final extension at 72 °C was maintained for 30 minutes, after which the reaction was cooled down to 12 °C. The PCR amplicon was purified using the QIAquick PCR purification kit (Qiagen, Cat. No. 28104). First, 500 µl of PB buffer was added to the PCR reaction, and the mix was transferred to the column. After spin down, the flow through was discarded, and the column was washed with 750 µl of PE buffer. The flow-through was removed again, and the column spun for 2 more minutes. The amplicon was eluted in 30 µl of elution buffer.

The amplicons were then used as templates for in vitro transcription. The reaction contained 6 µl of water, 2 µl of 10× reaction buffer, 2 µl of ATP, CTP, UTP, and GTP (100 mM each), 2 µl of T7 RNA polymerase mix (NEB, Cat. No. E2040S), and 1 µg of the amplicon. The reaction was incubated for 2 hours at room temperature. To remove the amplicon, 20 µl of water, 5 µl of 10X Zymo DNase buffer, and 5 µl of Zymo DNase I were added to the in vitro transcription product, and the reaction was incubated for 15 minutes at room temperature. The Zymo kit (Zymo Research, Cat. No. R1013) column purification protocol was used to purify the RNA. First, 50 µl of RNA binding buffer and 50 µl of 100% ethanol were added to the DNase-treated in vitro transcribed RNA. The mix was then transferred to a column, and the RNA was bound to the column by spinning down for 30 seconds. The column was washed with 400 µl of RNA prep buffer, then with 700 µl of wash buffer, followed by a final wash with 400 µl of wash buffer. After removing the flow through, the column was centrifuged for an additional 2 minutes, and then the RNA was eluted with 30 µl of nuclease-free water.

For standards with poly(A) tails 16, 32, or 64 nucleotides long, the poly(A) tail was encoded on the barcoded oligos (Supplementary Table 8). To obtain spikes with longer poly(A) tails, the RNA was polyadenylated by incubating with the *E. coli* Poly(A) polymerase for different amounts of time to achieve different poly(A) tail lengths. The poly(A) polymerization mix consisted of 1× *E. coli* poly(A) polymerase reaction buffer, ATP 1 mM, 0.5 µl of *E. coli* Poly(A) polymerase (NEB, M0276S), and 10 µg of purified in vitro transcribed RNA in a total volume of 20 µl. The reaction was incubated for 10 to 30 minutes for the different barcodes to archive poly(A) tails of different lengths. The RNA was purified again using the Zymo kit column as described above, and an equimolar master mix of the different standards 50 ng/µl was prepared.

### Poly(A) tail purification

Viral RNA and host mRNA were purified from total RNA using Dynabeads™ Oligo(dT)_25_ beads (ThermoFisher, Cat. No. 61002). To prepare the oligo(dT) beads, 20 µl of beads per sample were transferred to a low-binding tube and placed on a magnet to remove the storage buffer. The beads were washed with 40 µl of binding buffer (20 mM Tris-HCl pH 7.5, 1.0 M LiCl, and 2 mM EDTA) and resuspended in 40 µl of binding buffer. Then 5 µg of total RNA was diluted with nuclease-free water up to 40 µl and mixed with 40 µl of binding buffer, except for the virion where 1 µg of RNA was used. Diluted RNA was denatured by incubating at 65 °C for 2 minutes and immediately placed on ice. The denatured RNA was mixed with 40 µl of pre-equilibrated oligo(dT) beads and incubated for 5 minutes at room temperature. The supernatant was removed from the beads by incubating them on a magnetic rack for 5 minutes. The beads were washed twice with wash buffer (10 mM Tris-HCl, pH 7.5, 0.15 M LiCl, and 1 mM EDTA). After final washing, the supernatant was removed, and beads were resuspended in 10 µl of elution buffer (10 mM Tris-HCl, pH 7.5). The enriched mRNA was eluted by incubating at 80 °C for 2 minutes, and beads were separated using a magnetic stand.

### Preparation of barcoded oligos mix to capture terminal modifications

A 100 nM mixture of different barcoded oligos was made to detect tail modifications. First, ten sets of 1 µM annealed upper and lower strand oligos were made as listed in Supplementary Table 9. We incubated the annealing reaction at 94 °C for 5 minutes and slowly cooled down to room temperature in annealing buffer (0.01 M Tris-HCl pH 7.5, and 0.05 M NaCl). A 100 nM barcoded oligos mixture was prepared according to different ratios of A, U, and G capturing oligos as indicated in Supplementary Table 9. Oligo 1, identifiable with barcode 1, was used to capture non-modified poly(A) tails. Oligo 2, identifiable with barcode 2, was used to capture terminal mono-uridylation, while Oligos 3 to 6, identifiable with barcode 3, were used to capture terminal oligo-uridylation. Oligos 7 to 10, identifiable with barcode 4, were used to detect terminal guanylation.

### Direct RNA-seq

To detect the terminal RNA modifications, we generated direct RNA sequencing libraries using the protocol from Oxford Nanopore Technologies (Direct RNA Sequencing Kit- SQK-RNA002) with some minor changes to assemble the barcoded oligos that bind to the specific 3′ terminal nucleotides. A splint ligation was used to bind the RNA to the 3′ adaptors. For this, 3 µl of ligation buffer (6% PEG8000 in NEB 10× T4 DNA ligation buffer), 1.5 µl of T4 DNA ligase (NEB, Cat. No. M0202L), 1 µl of 0.1 ng/µl RNA spike mix, and 1.5 µl of barcoded oligos mix (100 nM) (Supplementary Table 9) were mixed with ~8 µl of poly(A) selected RNA in a 15 µl final volume. The reaction was incubated for 60 minutes at room temperature for efficient ligation.

To maximize the throughput of RNA libraries, the ligated RNA was reverse transcribed. The 15 µl of splint ligated RNA was mixed with 9 µl of water, 2 µl of dNTPs (10 mM), 8 µl of 5× first-strand buffer, 4 µl of 100 mM DTT, and 2 µl of SuperScript II reverse transcriptase (ThermoFisher, Cat. No. 18064022). The reaction was then incubated at 50 °C for 50 minutes, followed by 10 minutes at 70 °C, and cooled at 4 °C.

The resulting cDNA/RNA hybrid was purified using AMPure beads (Beckman Coulter, Cat. No. A63880). First, 72 µl of AMPure beads were added to each reaction and incubated at room temperature for 5 minutes. The supernatant was then removed using a magnetic stand, and beads were washed with 150 µl of 70% ethanol by rotating the tubes twice 180° on the magnetic stand. The beads were resuspended in 20 µl of nuclease-free water and incubated at room temperature for 5 minutes. The eluate was separated from beads using the magnetic stand.

The Nanopore adaptors with the motor protein (RMX) supplied with the Direct RNA Sequencing Kit (SQK-RNA002) were then ligated to the samples. The 20 µl of AMPure beads purified cDNA/RNA hybrid was mixed with 8 µl of ligation buffer (6% PEG8000 in NEB 10× T4 DNA ligation buffer), 6 µl of RMX-RNA adapter, 3 µl of nuclease-free water, and 3 µl of T4 DNA Ligase (NEB, Cat. No. M0202L) for 40 µl of the total final volume. The reaction was incubated for 15 minutes at room temperature, and then 40 µl of AMPure beads were mixed with adaptor-ligated RNA and incubated at room temperature for 5 minutes. The supernatant was removed using a magnetic stand, and beads were washed twice with 150 µl of wash buffer (WSB from SQK-RNA002). After washing, beads were resuspended in 21 µl of elution buffer (EB from SQK-RNA002) and incubated at room temperature for 20 minutes. The eluate was separated from beads using a magnetic stand and kept aside.

The libraries were prepared for loading into the Nanopore flow cells (R9.4.1) using the flow cell priming kit (EXP-FLP002) according to the manufacturer’s instructions. Briefly, 17.5 µl of nuclease-free water and 37.5 µl of RNA running buffer (RRB from SQK-RNA002) were added to the 21 µl of adaptor-ligated RNA samples and mixed well to prepare the loading mix. To prepare the flow cell, 30 µl of FLT solution was added to 1 ml of FB solution (from EXP-FLP002) at room temperature, and 800 µl of this solution was loaded to the flow cell through the priming port and incubated at room temperature for 5 minutes. Then, the spot-on port was gently opened, and 200 µl of the FLT-FB mix was loaded through the priming port. Finally, 75 µl of RNA library was loaded through the spot-on port, and both ports were closed after loading the library. Fast5 files were generated by the Nanopore MinKNOW software.

### RNA circularization assay

Circular RT-PCR was performed to determine the terminal modifications of the MHV virion genome following previously described protocols with some modifications^2,25^. Briefly, 1.0 µg of MHV virion RNA was used as a starting material and was subjected to decapping using 5U of mRNA decapping enzyme (NEB, Cat. No. M0608S) and incubated at 37 °C for 1 hour in a 20 µl reaction. After incubation, RNA was purified using RNA Clean & Concentrator-25 kit (Zymo, Cat. No. R1017) and eluted in 30 µl nuclease-free water. RNA was denatured at 95 °C for 5 minutes and quickly cooled on ice. Then 30 µl of decapped RNA was head to tail ligated using 10U of T4 RNA ligase I (NEB, Cat. No. M0204S) in 60 µl reaction containing 1× T4 RNA ligase I buffer, 1 mM ATP, 40U of RNase inhibitor, and 10% PEG8000 and incubated for 16 hours at 16 °C. After incubation, circular RNA was purified using RNA Clean & Concentrator-25 kit and resuspended in 30 µl nuclease-free water. Next, 15 µl of purified, circular RNA was reverse transcribed using SuperScript II reverse transcriptase (ThermoFisher, Cat. No. 18064022) with a gene-specific primer (5′-GTACGTACGGACGCCAATC-3′). The resulting circular cDNA was used to amplify the region spanning the poly(A) tail and terminal modifications of the MHV genome using AccuPrime Taq DNA polymerase (ThermoFisher, Cat. No. 12346086). To this end, 5 µl of cDNA was used in a PCR reaction with forward primer (5′-GATAGAGAAGGTTGTGGCAGAC-3′) and reverse primer (5′-GCAGAGAACGAAAGTCAAGGA-3′). The reaction was heated at 94 °C for 3 min, followed by 35 cycles of 30 seconds at 94 °C, 30 seconds at 57.5 °C and 1 minute at 72 °C and a final extension at 72 °C for 7 minutes. The PCR product was purified using a QIAquick PCR purification kit (Qiagen, Cat. No. 28104). The resulting PCR amplicon was ligated into the PCR 2.1 vector using a Topo cloning kit (ThermoFisher, Cat. No. 451641), and the reaction was incubated at room temperature for 1 hour. The ligated reaction was transformed into OneShot TOP10 competent cells and plated onto ampicillins/X-gal containing LB-plates. White colonies were screened by Sanger sequencing.

### Data analysis

Direct RNA-seq libraries were demultiplexed with DeePlexiCon version 1.2.0^26^ and base called with Guppy version 5.0.10. The demultiplexed reads were mapped to the mouse transcriptome and the MHV genome using Minimap2 version 2.20^27^. The poly(A) tail length was determined using Nanopolish^28^ version 0.13.3, and the terminal modifications were assigned according to the barcodes associated with each modification. The poly(A) tail frequency plots showing uridylation levels were obtained by normalizing the number of uridylated reads of any given length by the total number of reads, in contrast to only the total number of uridylated reads. This allows a direct comparison of the frequency of uridylated transcripts and all transcripts at each poly(A) tail length across plots. Differential gene expression was determined using DESeq2^29^. Only transcripts with 180 reads or more across the 18 samples were considered for analysis. The ontology of the differentially regulated genes was obtained using the TopGO R package. Pathway analysis of differentially expressed genes at 0 hpi and 24 hpi was performed using Ingenuity Pathway Analysis (IPA) (http://www.ingenuity.com/index.html). The read count matrix file obtained from DESeq2 was used as an input for core analysis. The graphical summaries of the pathway obtained from IPA were exported and manually curated using Inkscape. Measurements in every case were taken from distinct samples.

### Statistics and reproducibility

For the statistical data analysis of qPCR data, a one-way ANOVA with multiple comparison tests was performed using GraphPad Prism version 8.2.1. Four replicates were used for each condition. For the plaque assay time course between 8 and 48 hours, samples were taken from infections of different plates/wells (biological replicates). To control for batch effects, infections were performed on three different days, with 2–3 infections each day. For each biological replicate, we performed two technical replicates. The average of the technical replicates for each biological replicate was used for the statistical analysis. A similar strategy was used for the time course between 24 and 48 hours, this time using four biological replicates. The data were log_10_-transformed, and a two-way ANOVA with multiple comparison tests was performed for the statistical analysis using GraphPad Prism version 8.2.1. For DESeq differential gene expression analysis, three biological replicates were used. Transcripts with adjusted *p* values lower than 0.05 were considered differentially expressed.

### Reporting summary

Further information on research design is available in the Nature Portfolio Reporting Summary linked to this article.

## Supplementary information

Supplementary Information

Description of Additional Supplementary Files

Supplementary Data 1

Reporting Summary

## Data Availability

The direct RNA sequencing data have been deposited at the Gene Expression Omnibus (GEO)^30^ with accession number GSE200416. Raw data for the plaque assay is provided in Supplementary Data 1. Other data are available from the corresponding author on reasonable request.
