# Supplementary material for: TUT4/7-mediated uridylation of a coronavirus subgenomic RNAs delays viral replication
Source: Commun Biol. 2023 Apr 21;6:438. doi: 10.1038/s42003-023-04814-1 (PMC10119532; doi:10.1038/s42003-023-04814-1)
Supplement: Supplementary file 1 — Supplementary Information [file 42003_2023_4814_MOESM1_ESM.pdf]

Supplementary Information

Supplementary Tables

Supplementary Table 1. Number (and percentage) of MHV-mapping reads guanylated, uridylated, mono- and oligo-uridylated in each Nanopore sequencing library. All reads passed the poly(A) tail calling quality control and had a confidence interval >0.95 for barcode assignment.

				Reads					
Cell type	Condition	hpi	Replicate	Total	Not modified	Uridylated	Mono-uridylated	Oligo-uridylated	Guanylated
17-CL1	shCTL	24	1	117658	98566 (83.77)	18024 (15.32)	13952 (11.86)	4072 (3.46)	1068 (0.91)
17-CL1	shCTL	24	2	162052	139117 (85.85)	21427 (13.22)	17441 (10.76)	3986 (2.46)	1508 (0.93)
17-CL1	shCTL	24	3	157142	135221 (86.05)	20617 (13.12)	16653 (10.6)	3964 (2.52)	1304 (0.83)
17-CL1	shTUT47	24	1	36074	31329 (86.85)	4459 (12.36)	3481 (9.65)	978 (2.71)	286 (0.79)
17-CL1	shTUT47	24	2	85240	76500 (89.75)	7957 (9.33)	6687 (7.84)	1270 (1.49)	783 (0.92)
17-CL1	shTUT47	24	3	37730	33766 (89.49)	3728 (9.88)	3181 (8.43)	547 (1.45)	236 (0.63)
17-CL1	shCTL	48	1	26239	24529 (93.48)	1281 (4.88)	1100 (4.19)	181 (0.69)	429 (1.63)
17-CL1	shCTL	48	2	75780	70161 (92.59)	4770 (6.30)	4060 (5.36)	710 (0.94)	849 (1.12)
17-CL1	shCTL	48	3	34228	32033 (93.59)	1661 (4.85)	1438 (4.20)	223 (0.65)	534 (1.56)
17-CL1	shTUT47	48	1	258451	227285 (87.94)	29145 (11.27)	23194 (8.97)	5951 (2.30)	2021 (0.78)
17-CL1	shTUT47	48	2	203310	175061 (86.11)	26798 (13.18)	21218 (10.44)	5580 (2.74)	1451 (0.71)
17-CL1	shTUT47	48	3	380401	322079 (84.67)	54610 (14.35)	43491 (11.43)	11119 (2.92)	3712 (0.98)
17-CL1	WT	24	1	34771	31525 (90.66)	2975 (8.56)	2464 (7.09)	511 (1.47)	271 (0.78)
17-CL1	WT	24	2	32435	29312 (90.37)	2885 (8.89)	2398 (7.39)	487 (1.50)	238 (0.73)
17-CL1	WT	24	3	36368	32792 (90.17)	3276 (9.01)	2725 (7.49)	551 (1.52)	300 (0.82)
17-CL1	WT	48	1	98852	89826 (90.87)	8255 (8.35)	6633 (6.71)	1622 (1.64)	771 (0.78)
17-CL1	WT	48	2	80141	72609 (90.60)	6944 (8.67)	5542 (6.92)	1402 (1.75)	588 (0.73)
17-CL1	WT	48	3	52838	47684 (90.25)	4759 (9.01)	3777 (7.15)	982 (1.86)	395 (0.75)
NCTC	WT	24	1	141851	116199 (81.92)	24952 (17.59)	20639 (14.55)	4313 (3.04)	700 (0.49)
NCTC	WT	24	2	110846	91662 (82.69)	18534 (16.72)	13941 (12.58)	4593 (4.14)	650 (0.59)
-	-	virion	1	148636	132803 (89.35)	14673 (9.87)	12711 (8.55)	1962 (1.32)	1160 (0.78)
-	-	virion	2	139067	124018 (89.18)	13870 (9.97)	11695 (8.41)	2175 (1.56)	1179 (0.85)

Supplementary Table 2. Number of total reads, number of reads mapped to the MHV genome, number of reads mapped to endogenous transcripts, number of detected transcripts, and number of detected genes.

				Reads				
Cell type	Condition	hpi	Replicate	Total	MHV	Endogenous	Transcripts	Genes
17-CL1	shCTL	0	1	646,457	-	639,974	22,577	11,215
17-CL1	shCTL	0	2	523,601	-	518,899	21,002	10,559
17-CL1	shCTL	0	3	643,288	-	636,008	23,287	10,868
17-CL1	shTUT47	0	1	646,176	-	639,445	22,110	10,722
17-CL1	shTUT47	0	2	548,125	-	540,775	21,394	10,446
17-CL1	shTUT47	0	3	537,496	-	530,784	20,865	10,463
17-CL1	shCTL	24	1	592,193	217,578	369,580	20,051	10,476
17-CL1	shCTL	24	2	612,319	273,647	332,700	18,742	10,167
17-CL1	shCTL	24	3	611,246	273,174	332,919	19,123	10,268
17-CL1	shTUT47	24	1	673,470	65,632	601,213	20,972	10,774
17-CL1	shTUT47	24	2	924,736	140,970	774,977	22,297	11,107
17-CL1	shTUT47	24	3	747,530	56,592	683,264	20,814	10,684
17-CL1	shCTL	48	1	469,118	48,015	415,170	19,938	10,674
17-CL1	shCTL	48	2	583,332	136,133	441,612	21,384	11,183
17-CL1	shCTL	48	3	526,799	59,896	458,677	21,307	10,944
17-CL1	shTUT47	48	1	647,672	416,160	227,937	14,892	9,124
17-CL1	shTUT47	48	2	562,834	356,786	203,603	13,997	8,851
17-CL1	shTUT47	48	3	1,012,718	666,790	341,599	18,640	10,295
17-CL1	WT	24	1	617,070	55,983	555,938	21,908	10,769
17-CL1	WT	24	2	597,511	53,984	538,347	21,751	10,804
17-CL1	WT	24	3	603,117	59,382	538,849	21,359	10,761
17-CL1	WT	48	1	594,742	170,869	413,909	21,670	11,024
17-CL1	WT	48	2	555,065	148,276	397,583	21,718	10,964
17-CL1	WT	48	3	391,943	96,738	289,102	19,542	10,524
NCTC	WT	24	1	434,343	257,502	173,076	16,123	9,478
NCTC	WT	24	2	308,652	181,122	123,963	14,429	8,966
-	-	virion	1	253,611	250,701	2,631	1,140	1,000
-	-	virion	2	274,548	272,224	2,092	946	817

Supplementary Table 3. 3' terminal sequences from MHV virion RNAs obtained by RNA circularization. Clones from two independent replicates are shown. Terminal modifications to the poly(A) tail are highlighted in red.

Replicate	Colony	Sequences
1	1	CATGGCCAATTGGAAGAATCACAAAAAAAAAAAAAAAAAAAAAAAAAAAAAAAAAAAAAAAAAAAA<br>AAAAAAAAAAAAAAAAAAAAAAAAAAA
1	2	CATGGCCAATTGGAAGAATCACAAAAAAAAAAAAAAAAAAAAAAAAAAAAAAAAAAAAAAAAAAAA<br>AAAAAAAAA
1	3	CATGGCCAATTGGAAGAATCACAAAAAAAAAAAAAAAAAAAAAAAAAAAAAAAAAAAAAAAAAAAA<br>AAA
1	4	CATGGCCAATTGGAAGAATCACAAAAAAAAAAAAAAAAAAAAAAAAAAAAAAAAAAAAAAAAAAAA<br>AA
1	5	CATGGCCAATTGGAAGAATCACAAAAAAAAAAAAAAAAAAAAAAAAAAAAAAAAAAAA
1	6	CATGGCCAATTGGAAGAATCACAAAAAAAAAAAAAAAAAAAAAAAAAAAAAAAAAAA
1	7	CATGGCCAATTGGAAGAATCACAAAAAAAAAAAAAAAAAAAAAAAAAAAAAAA**TTT**
1	8	CATGGCCAATTGGAAGAATCACAAAAAAAAAAAAAAAAAAAAAAAAAAA
1	9	CATGGCCAATTGGAAGAATCACAAAAAAAAAAAAAAAAAAAAAAAA**TT**
1	10	CATGGCCAATAGGAAGAATCACAAAAAAAAAAAAAAAAAAAAAAA
1	11	CATGGCCAATTGGAAGAATCACAAAAAAAAAAAAAAAAAAA
1	12	CATGGCCAATTGGAAGAATCACAAAAAAAAAAAAAAAAAA
1	13	CATGGCCAATTGGAAGAATCACAAAAAAAAAAAAAAAAA
1	14	CATGGCCAATTGGAAGAATCACAAAAAAAAAAAAAAAA**T**
1	15	CATGGCCAATTGGAAGAATCACAAAAAAAAAAAAAA
1	16	CATGGCCAATTGGAAGAATCACAAAAAAAAAAAAAA
2	1	CNNGNCCAATTGGAAGANTCNCAAAAAAAAAAAAAAAAAAAAAAAAAAAAAAAAAAAAAAAAAAAA
2	2	NNGNCCANTNGGAAGAATCNCAAAAAAAAAAAAAAAAAAAAAAAAAAAAAAAAAAAAAAA
2	3	CATGGCCAATTGGAAGAATCACAAAAAAAAAAAAAAAAAAAAAAAAAAA
2	4	CATGGCCAATTGGAAGAATCACAAAAAAAAAAAAAAAAAAAAAAAAA**TT**
2	5	CATGGCCAATTGGAAGAATCACAAAAAAAAAAAAAAAAAAAAAAA**TTT**
2	6	CATGGCCAATTGGAAGAATCACAAAAAAAAAAAAAAAAAAAAAAA
2	7	CATGGCCAATTGGAAGAATCACAAAAAAAAAAAAAA**TTTTTA**
2	8	CATGGCCAATTGGAAGAATCACAAAAAAAAAAAAAAAAAAAA
2	9	CATGGCCAATAGGAAGAATCACAAAAAAAAAAAAAAAAAA
2	10	CATGGCCAATTGGAAGAATCACAAAAAAAAAAAAAAAAA
2	11	CATGGCCAATTGGAAGAATCACAAAAAAAAAAAAAAAA
2	12	CATGGCCAATTGGAAGAATCACAAAAAAAAAAAAAAA

Supplementary Table 4. Mean percentages of non-modified poly(A) tails, mono-uridylated tails, oligo-uridylated tails, and guanylated tails for the different libraries generated split by MHV RNA species.

			% of reads				
**Condition**	**hpi**	**sgRNA**	**Not modified**	**Uridylated**	**Mono-uridylated**	**Oligo-uridylated**	**Guanylated**
shCTL	24	E	85.0	14.0	11.2	2.8	1.0
shCTL	24	M	83.1	16.0	12.8	3.2	0.9
shCTL	24	N	85.5	13.5	10.3	3.3	1.0
shCTL	24	gRNA/S	86.6	12.4	10.2	2.2	1.0
shCTL	48	E	92.2	6.3	5.3	1.0	1.5
shCTL	48	M	91.2	7.2	6.2	1.1	1.6
shCTL	48	N	93.1	5.3	4.3	1.0	1.6
shCTL	48	gRNA/S	93.8	4.7	3.9	0.8	1.6
shTUT47	24	E	88.4	10.8	8.7	2.1	0.8
shTUT47	24	M	87.5	11.7	9.7	2.0	0.9
shTUT47	24	N	88.5	10.6	8.2	2.4	0.9
shTUT47	24	gRNA/S	90.2	9.3	7.7	1.6	0.6
shTUT47	48	E	84.5	14.6	11.6	3.0	1.0
shTUT47	48	M	85.0	14.1	11.2	2.9	1.0
shTUT47	48	N	86.4	12.7	9.5	3.2	0.9
shTUT47	48	gRNA/S	86.3	12.8	10.1	2.7	0.9
WT_24	24	E	90.0	9.2	7.5	1.7	0.8
WT_24	24	M	88.6	10.5	8.7	1.8	0.9
WT_24	24	N	90.4	8.8	6.9	1.9	0.8
WT_24	24	gRNA/S	91.8	7.6	6.0	1.5	0.6
WT_48	48	E	89.4	9.7	7.8	2.0	0.8
WT_48	48	M	88.4	10.9	8.7	2.2	0.7
WT_48	48	N	90.5	8.6	6.5	2.1	0.9
WT_48	48	gRNA/S	91.1	8.2	6.6	1.5	0.7

Supplementary Table 5. Number (and percentage) of guanylated, uridylated, mono- and oligo-uridylated reads mapping to endogenous transcripts in each Nanopore sequencing library. All reads passed the poly(A) tail calling quality control and had a confidence interval >0.95 for barcode assignment.

				Reads					
**Cell type**	**Condition**	**hpi**	**Replicate**	**Total**	**Not modified**	**Uridylated**	**Mono-uridylated**	**Oligo-uridylated**	**Guanylated**
17-CL1	shCTL	0	1	391902	374200 (95.48)	12594 (3.22)	9900 (2.53)	2694 (0.69)	5108 (1.30)
17-CL1	shCTL	0	2	314470	300820 (95.66)	9646 (3.07)	7489 (2.38)	2157 (0.69)	4004 (1.27)
17-CL1	shCTL	0	3	388828	373097 (95.95)	11662 (3.00)	8946 (2.30)	2716 (0.70)	4069 (1.05)
17-CL1	shTUT47	0	1	397321	383328 (96.48)	10521 (2.65)	8217 (2.07)	2304 (0.58)	3472 (0.87)
17-CL1	shTUT47	0	2	329734	314863 (95.49)	10214 (3.10)	8104 (2.46)	2110 (0.64)	4657 (1.41)
17-CL1	shTUT47	0	3	326677	314084 (96.15)	8875 (2.71)	6808 (2.08)	2067 (0.63)	3718 (1.14)
17-CL1	shCTL	24	1	207615	197319 (95.04)	7884 (3.80)	5897 (2.84)	1987 (0.96)	2412 (1.16)
17-CL1	shCTL	24	2	201026	191383 (95.20)	7441 (3.70)	5919 (2.94)	1522 (0.76)	2202 (1.10)
17-CL1	shCTL	24	3	195958	186586 (95.22)	7196 (3.67)	5640 (2.88)	1556 (0.79)	2176 (1.11)
17-CL1	shTUT47	24	1	345721	331209 (95.80)	11083 (3.20)	8480 (2.45)	2603 (0.75)	3429 (0.99)
17-CL1	shTUT47	24	2	490793	472195 (96.21)	13529 (2.76)	10642 (2.17)	2887 (0.59)	5069 (1.03)
17-CL1	shTUT47	24	3	448858	432904 (96.45)	12396 (2.76)	9828 (2.19)	2568 (0.57)	3558 (0.79)
17-CL1	shCTL	48	1	240297	228983 (95.29)	7261 (3.03)	5614 (2.34)	1647 (0.69)	4053 (1.69)
17-CL1	shCTL	48	2	262037	252650 (96.42)	6673 (2.55)	5056 (1.93)	1617 (0.62)	2714 (1.04)
17-CL1	shCTL	48	3	275316	263304 (95.64)	7711 (2.80)	5977 (2.17)	1734 (0.63)	4301 (1.56)
17-CL1	shTUT47	48	1	143479	139176 (97.00)	3093 (2.16)	2194 (1.53)	899 (0.63)	1210 (0.84)
17-CL1	shTUT47	48	2	121456	117493 (96.74)	2918 (2.41)	1986 (1.64)	932 (0.77)	1045 (0.86)
17-CL1	shTUT47	48	3	208083	199547 (95.90)	6338 (3.05)	4263 (2.05)	2075 (1.00)	2198 (1.06)
17-CL1	WT	24	1	343472	326936 (95.19)	13306 (3.87)	10559 (3.07)	2747 (0.80)	3230 (0.94)
17-CL1	WT	24	2	323707	308026 (95.16)	12592 (3.89)	9880 (3.05)	2712 (0.84)	3089 (0.95)
17-CL1	WT	24	3	333127	317163 (95.21)	12805 (3.84)	10069 (3.02)	2736 (0.82)	3159 (0.95)
17-CL1	WT	48	1	248190	237404 (95.65)	8769 (3.53)	6796 (2.74)	1973 (0.79)	2017 (0.81)
17-CL1	WT	48	2	228246	217702 (95.38)	8504 (3.72)	6462 (2.83)	2042 (0.89)	2040 (0.89)
17-CL1	WT	48	3	171741	163958 (95.47)	6351 (3.70)	4739 (2.76)	1612 (0.94)	1432 (0.83)
NCTC	WT	24	1	88783	83573 (94.13)	4470 (5.03)	3297 (3.71)	1173 (1.32)	740 (0.83)
NCTC	WT	24	2	68508	64855 (94.67)	3032 (4.43)	2219 (3.24)	813 (1.19)	621 (0.91)
-	-	virion	1	1471	1375 (93.47)	88 (5.98)	75 (5.10)	13 (0.88)	8 (0.54)
-	-	virion	2	1032	966 (93.60)	55 (5.33)	42 (4.07)	13 (1.26)	11 (1.07)

Supplementary Table 6. shRNA vectors for generating 17-CL1 lentiviral stable cell lines.

**Cell line name**	**Known-down target gene**	**shRNA sense sequences**
17-CL1 shCTL	Non-target	shCTL: ATCTCGCTTGGGCGAGAGTAAG
17-CL1 shTUT4/7	*Tut4* and *Tut7*	shTut4: GAGGAAACGTGTCCGAGTA<br>shTut7: AGGATTTTCCAGGAACTAA

Supplementary Table 7. qPCR primers.

**Gene ID**	**Forward primer**	**Reverse primer**
Tut4	AAGTCAGAAATTGGGACCAGC	TGGCAGCGTTTACTTTACATGAT
Tut7	TGGTCTGGGAATACACTGACA	AACTCTAAAGCATAGAACCGCAG
Gapdh	AATGGATTTGGACGCATTGGT	TTTGCACTGGTACGTGTTGAT

Supplementary Table 8. Oligos used to generate spike-ins of different poly(A) tail lengths.

**Oligo name**	**Oligo sequence**
RNA-Spike_Frw	CGTCGAGGAGTAATACGACTCACTATAGAATCCTGGCCCAGTGAGCAA
RNA-Spike_8	T(8)GGCACAGTCGGCACATACACGCTCACAGGCTGATCAGCGAGCTCTA
RNA-Spike_16	T(16)GGCACAGTCGGCTCGTCGCGCGCACAAGGCTGATCAGCGAGCTCTA
RNA-Spike_32	T(32)GGCACAGTCGACAGTGCGCTGTCTATAGGCTGATCAGCGAGCTCTA
RNA-Spike_64_a	T(64)GGCACAGTCGTCACACTCTAGAGCGAAGGCTGATCAGCGAGCTCTA
RNA-Spike_64_b	GGCACAGTCGCGCTGCGAGAGACAGTAGGCTGATCAGCGAGCTCTA
RNA-Spike_100	GGCACAGTCGATGACAGTGCTCAGTGAGGCTGATCAGCGAGCTCTA

Supplementary Table 9. Splint ligation oligos for direct RNA-seq library preparation.

**Oligo name**	**Oligo sequence**	**Barcode**	**Percent amount**
Oligo 1F	/5Phos/GGCTTCTTCTTGCTCTTAGGTAGTAGGTTC	BC-1	90%
Oligo 1R	GAGGCGAGCGGTCAATTTTCCTAAGAGCAAGAAGAAGCCTTTTT TTTTT		
Oligo 2F	/5Phos/GTGATTCTCGTCTTTCTGCGTAGTAGGTTC	BC-2	3%
Oligo 2R	GAGGCGAGCGGTCAATTTTCGCAGAAAGACGAGAATCACATTTT TTTTT		
Oligo 3F	/5Phos/GTACTTTTCTCTTTGCGCGGTAGTAGGTTC	BC-3	2.40%
Oligo 3R	GAGGCGAGCGGTCAATTTTCCGCGCAAAGAGAAAAGTACAATTT TTTTT		
Oligo 4F	/5Phos/GTACTTTTCTCTTTGCGCGGTAGTAGGTTC	BC-3	0.45%
Oligo 4R	GAGGCGAGCGGTCAATTTTCCGCGCAAAGAGAAAAGTACAAATT TTTTT		
Oligo 5F	/5Phos/GTACTTTTCTCTTTGCGCGGTAGTAGGTTC	BC-3	0.08%

Oligo 5R	GAGGCGAGCGGTCAATTTTCCGCGCAAAGAGAAAAGTACAAAATTTTTT		
Oligo 6F	/5Phos/GTACTTTTCTCTTTGCGCGGTAGTAGGTTC	BC-3	0.08%
Oligo 6R	GAGGCGAGCGGTCAATTTTCCGCGCAAAGAGAAAAGTACAAAAATTTTT		
Oligo 7F	/5Phos/GGTCTTCGCTCGGTCTTATTTAGTAGGTTC	BC-4	3%
Oligo 7R	GAGGCGAGCGGTCAATTTTAATAAGACCGAGCGAAGACCCTTTTTTTTT		
Oligo 8F	/5Phos/GGTCTTCGCTCGGTCTTATTTAGTAGGTTC	BC-4	0.75%
Oligo 8R	GAGGCGAGCGGTCAATTTTAATAAGACCGAGCGAAGACCTCTTTTTTTT		
Oligo 9F	/5Phos/GGTCTTCGCTCGGTCTTATTTAGTAGGTTC	BC-4	0.20%
Oligo 9R	GAGGCGAGCGGTCAATTTTAATAAGACCGAGCGAAGACCTTCTTTTTTT		
Oligo 10F	/5Phos/GGTCTTCGCTCGGTCTTATTTAGTAGGTTC	BC-4	0.05%
Oligo 10R	GAGGCGAGCGGTCAATTTTAATAAGACCGAGCGAAGACCTTTCTTTTTT		

Supplementary Figures

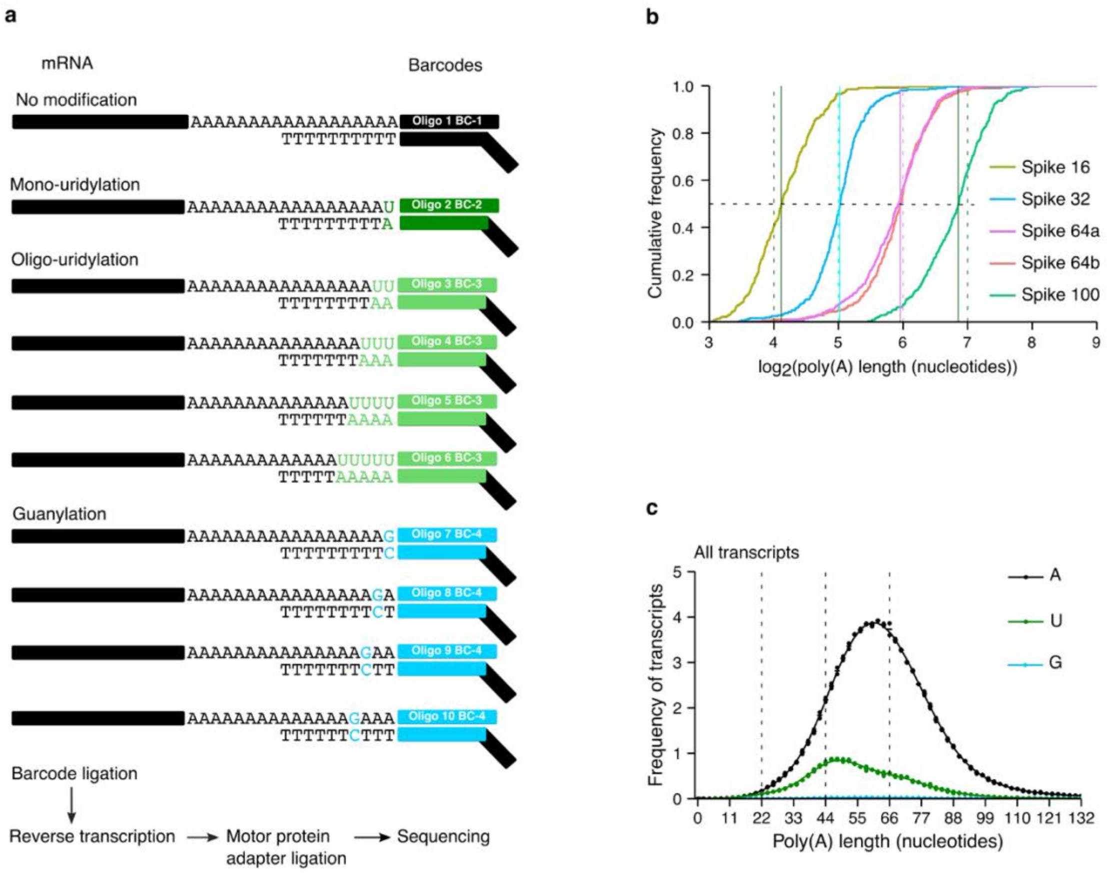

Supplementary Fig. 1. Calibration of the direct RNA sequencing protocol used to simultaneously measure poly(A) tail lengths and terminal modifications. (a) Depiction of the strategy used to capture terminal mono-uridylation, oligo-uridylation and guanylation. The capturing sequence and barcode assignation for each oligo used is shown. **(b)** A cumulative frequency plot for the poly(A) tail length for the different spikes is shown. **(c)** The poly(A) tail length profiles of NCTC cells infected with MHV at 24 hours post-infection (hpi) are shown in black for tails without terminal modifications, in green for terminally uridylated tails, and in cyan for terminally guanylated tails. Dots represent the frequency of poly(A) tail length for each sample, and the horizontal bars show the mean frequency of poly(A) tail length per condition (n = 2 for each condition).

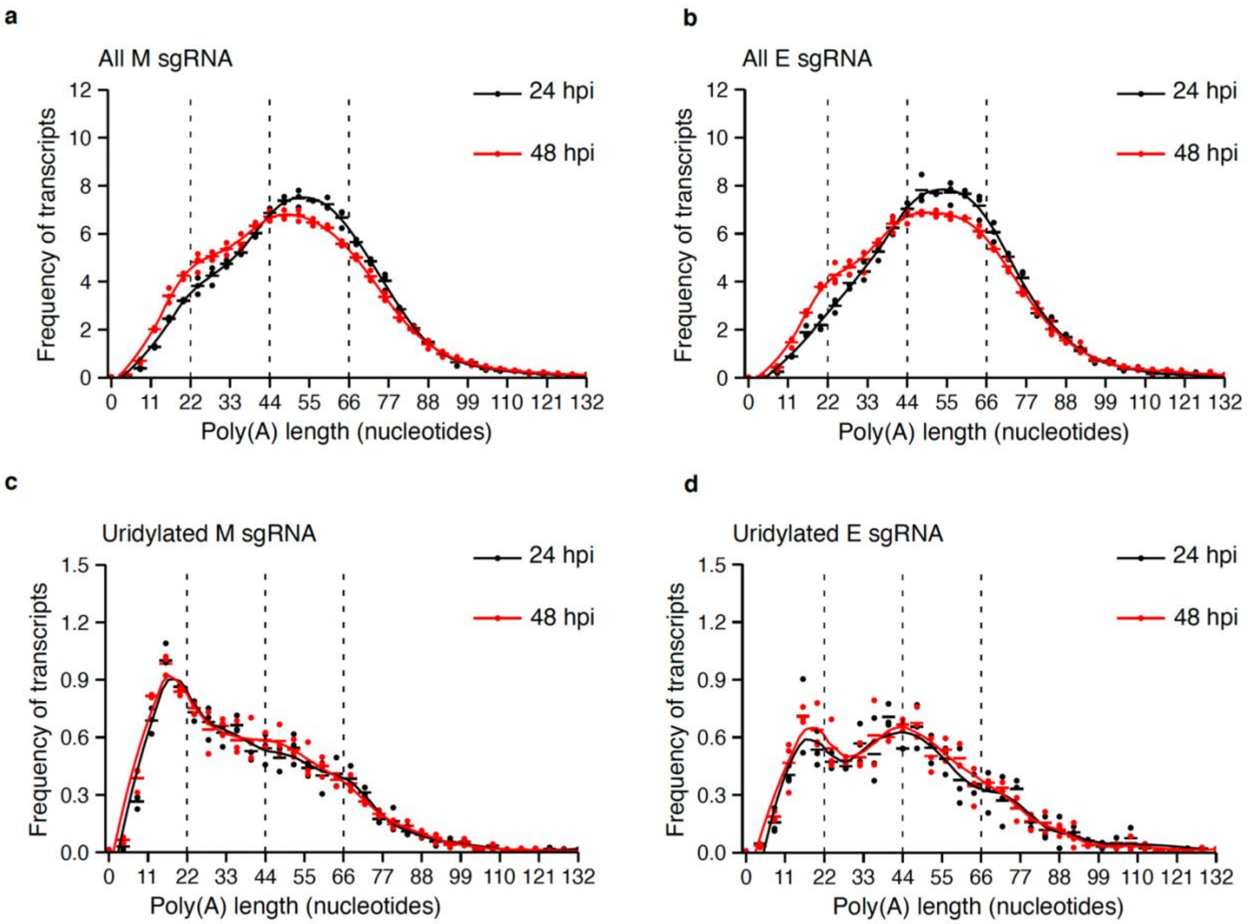

Supplementary Fig. 2. M and E sgRNAs with poly(A) tails shorter than ~22 nucleotides are uridylated. (a-b) The poly(A) tail length profiles of the M sgRNA **(a)** or E sgRNA **(b)** from 17-CL1 infected cells at 24 and 48 hours post-infection (hpi) are depicted in black and red, respectively. **(c)** The uridylation profiles of the M sgRNA from 17-CL1 infected cells at 24 hpi (black) and 48 hpi (red) are shown. Uridylated reads represent 10.5% and 10.9% of total reads at 24 hpi and 48 hpi, respectively. **(d)** The uridylation profiles of the E sgRNA from 17-CL1 infected cells at 24 hpi (black) and 48 hpi (red) are shown. Uridylated reads represent 9.2% and 9.7% of total reads at 24 hpi and 48 hpi, respectively. Dots represent the frequency of poly(A) tail length for each sample, and the horizontal bars show the mean frequency of poly(A) tail length per condition (n=3 for all conditions).

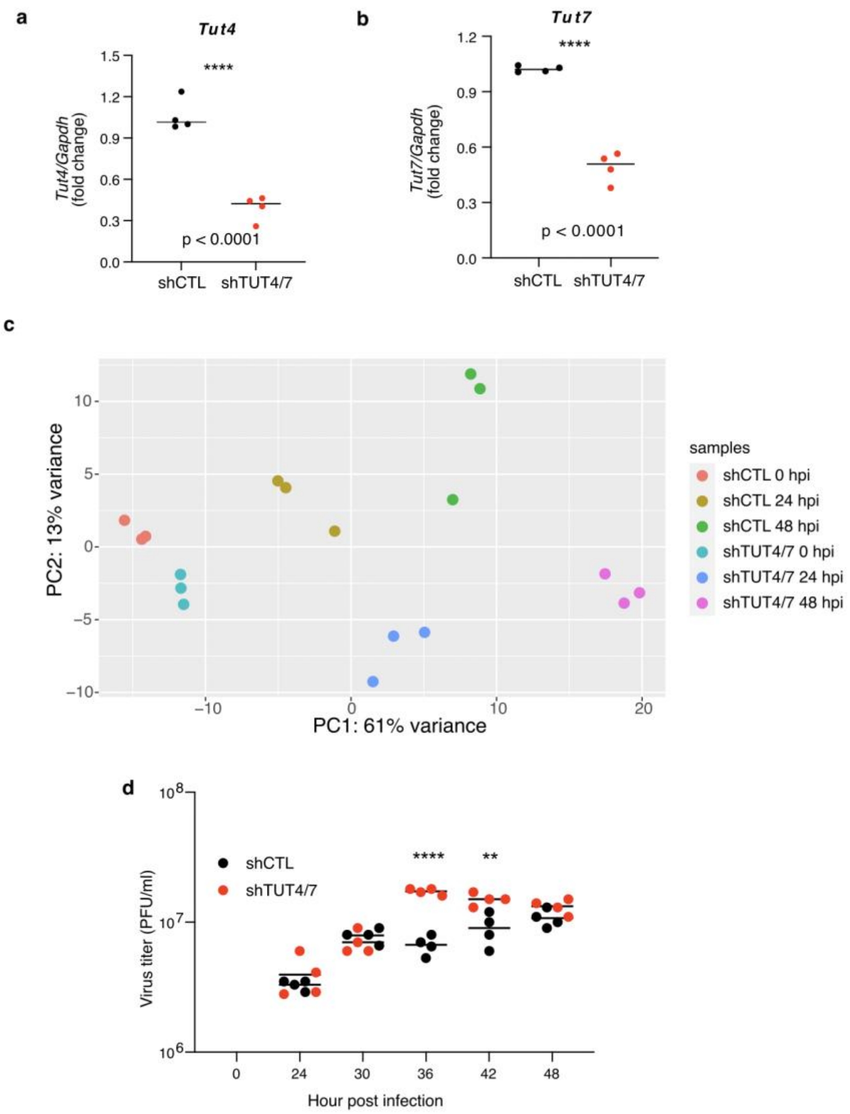

Supplementary Fig. 3. Transcriptomic signature of shCTL and shTUT4/7 cell lines before and during infection. (a, b) The relative expression of *Tut4* **(a)** and *Tut7* **(b)** transcripts between shCTL and shTUT4/7 cells as determined by qPCR is shown. Mouse *Gapdh* was used as an internal control. **(c)** A principal component analysis (PCA) plot from shCTL and shTUT4/7 triplicates before infection, at 24 hpi and 48 hours post-infection (hpi), is shown. Different samples are indicated in different colors. **(d)** The quantification of viral particles in the supernatant of MHV-infected shCTL and shTUT4/7 cells using plaque assay is shown for different time points (0, 24, 30, 36, 42, and 48 hpi). Virus quantification from shCTL and shTUT4/7 samples are indicated in black and red, respectively. Each dot represents one biological replicate (n = 4), and the bars indicate the mean value for each condition at every time point (Two-way ANOVA; ****, p<0.0001; ** p<0.01).

a

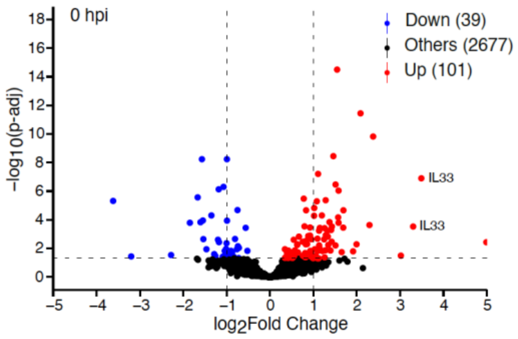

b

GO ID	GO Term	-log10p
GO:0051239	regulation of multicellular organismal process	9.89
GO:0009605	response to external stimulus	9.54
GO:0032101	regulation of response to external stimulus	8.00
GO:0097529	myeloid leukocyte migration	7.78
GO:0006954	inflammatory response	7.52
GO:0030595	leukocyte chemotaxis	7.07
GO:0032879	regulation of localization	7.03
GO:0001817	regulation of cytokine production	7.00
GO:0032103	positive regulation of response to external	6.92
GO:0051240	positive regulation of multicellular organism	6.92

c

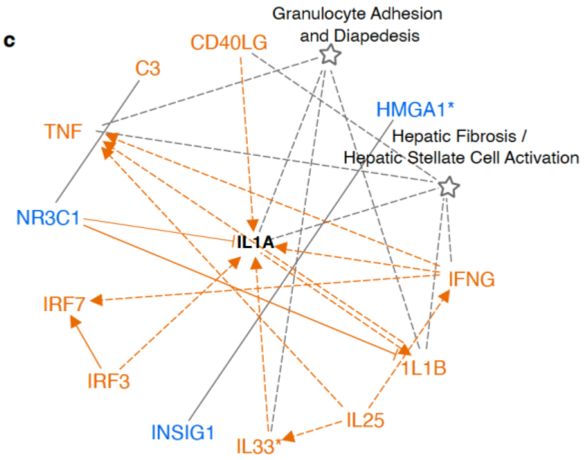

d

GO ID	GO Term	-log10p
GO:0006412	translation	21.45
GO:0043043	peptide biosynthetic process	21.06
GO:0006518	peptide metabolic process	20.55
GO:0043604	amide biosynthetic process	19.96
GO:0002181	cytoplasmic translation	19.16
GO:0043603	cellular amide metabolic process	18.07
GO:1901566	organonitrogen compound biosynthetic process	17.04
GO:0006090	pyruvate metabolic process	9.72
GO:0006096	glycolytic process	8.72
GO:0006757	ATP generation from ADP	8.72

e

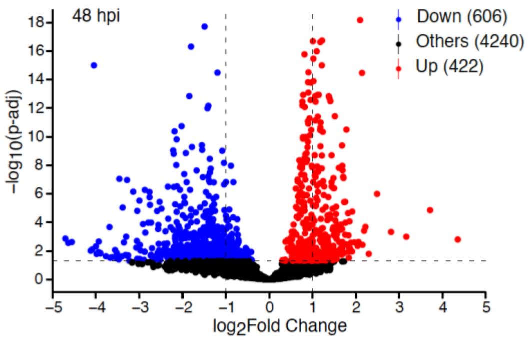

f

GO ID	GO Term	-log10p
GO:0043043	peptide biosynthetic process	25.15
GO:0006518	peptide metabolic process	24.92
GO:0006412	translation	24.85
GO:0043603	cellular amide metabolic process	23.72
GO:0043604	amide biosynthetic process	22.77
GO:0002181	cytoplasmic translation	20.89
GO:1901566	organonitrogen compound biosynthetic process	13.01
GO:0042255	ribosome assembly	10.55
GO:0042273	ribosomal large subunit biogenesis	9.36
GO:0042274	ribosomal small subunit biogenesis	7.77

Supplementary Fig. 4. Gene ontology and pathway analysis of differentially expressed transcripts between control and TUT4/7-depleted cells. (a) Volcano plot showing the differential gene expression between shCTL and shTUT4/7 cells before infection. Significantly up-regulated genes are indicated in red, and significantly down-regulated genes in blue (adjusted p-value < 0.05, n=3). **(b)** Table showing the ten most significantly enriched GO categories with

their GO IDs and p-values for upregulated transcripts in shTUT4/7 cells before infection. **(c)** A graphical summary of Ingenuity Pathway Analysis (IPA) of differentially expressed transcripts upon TUT4/7 depletion before infection is shown with upregulated pathways indicated in orange and downregulated in blue. Canonical pathways are indicated with stars. Direct and indirect interactions are indicated by full and dotted lines, respectively. **(d)** Table showing the ten most significantly enriched GO categories with their GO IDs and p-values for upregulated transcripts in shTUT4/7 cells at 24 hours post-infection (hpi) as in (b). **(e)** Volcano plot showing the differential gene expression between shCTL and shTUT4/7 cells at 48 hpi as in (a). **(f)** Table showing the ten most significantly enriched GO categories with their GO IDs and p-values for upregulated transcripts in shTUT4/7 cells at 48 hpi.

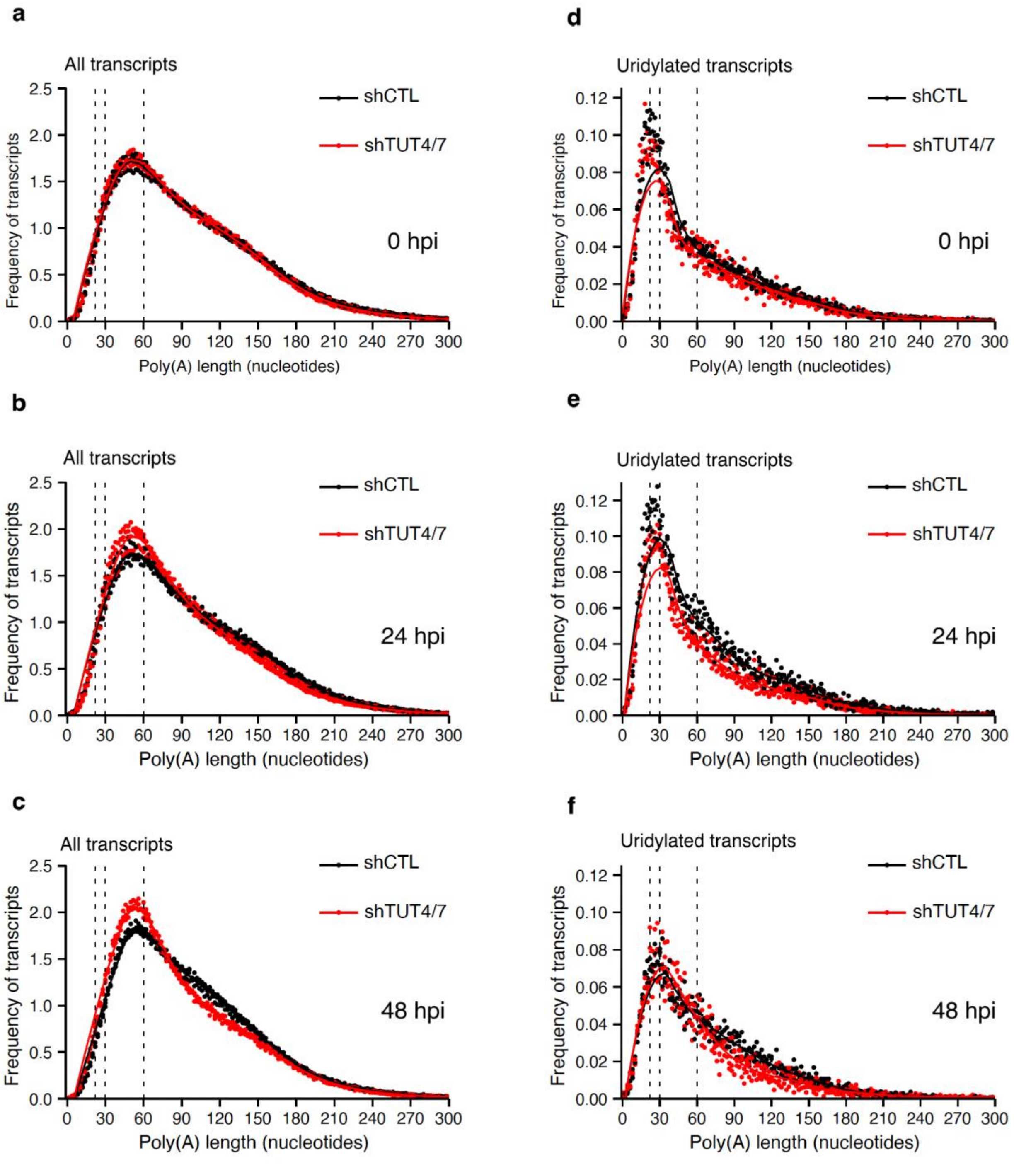

Supplementary Fig. 5. Poly(A) tail length profiles of all and terminal uridylated endogenous transcripts from shCTL and shTUT4/7 cell lines. (a, c) The poly(A) tail length profiles of endogenous transcripts from shCTL (black) and shTUT4/7 (red) cells before infection **(a)**, at 24 hours post-infection (hpi) **(b)** and 48 hpi **(c)**, are shown. **(d)** The poly(A) tail length profiles of terminally uridylated endogenous transcripts from shCTL (black) and shTUT4/7 (red) cells before

infection are shown. Uridylated reads represent 3.1% and 2.8% of total reads in shCTL and shTUT4/7 cells, respectively. **(e)** The poly(A) tail length profiles of terminally uridylated endogenous transcripts from shCTL (black) and shTUT4/7 (red) infected cells at 24 hpi are shown. Uridylated reads represent 3.7% and 2.9% of total reads in shCTL and shTUT4/7 cells, respectively. **(f)** The poly(A) tail length profiles of terminally uridylated endogenous transcripts from shCTL (black) and shTUT4/7 (red) infected cells at 48 hpi are shown. Uridylated reads represent 2.8% and 2.5% of total reads in shCTL and shTUT4/7 cells, respectively. Dots represent the frequency of poly(A) tail length for each sample, and the horizontal bars show the mean frequency of poly(A) tail length per condition (n=3 for all conditions).

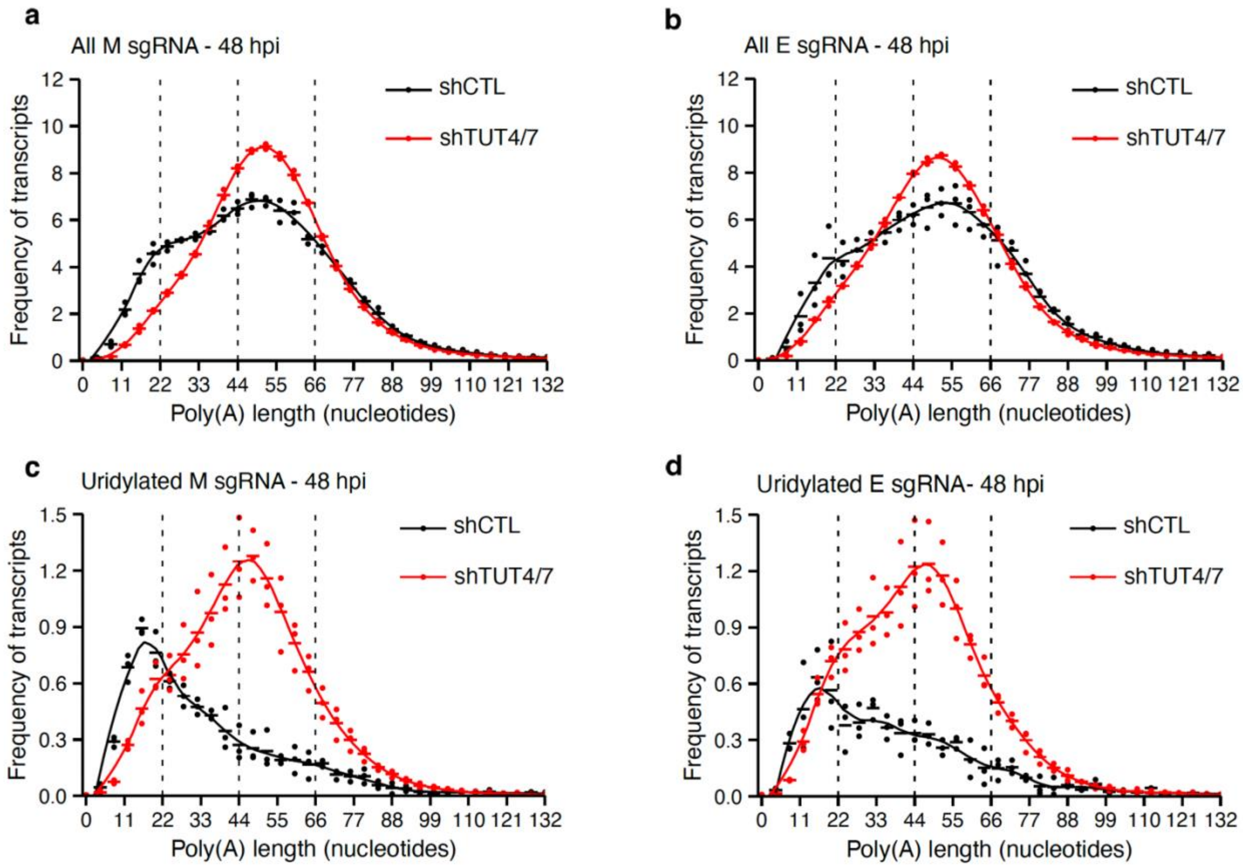

Supplementary Fig. 6. TUT4/7 alter the uridylation profiles of the M and E sgRNAs at 48 hpi. (a-b) The poly(A) tail profile of the M sgRNA **(a)** and E sgRNA **(b)** from shCTL (black) and shTUT4/7 (red) infected cells at 48 hours post-infection (hpi). **(c)** The poly(A) tail length profiles of terminally uridylated M sgRNA from shCTL (black) and shTUT4/7 (red) infected cells are shown. Uridylated reads represent 7.2% and 14.1% of total reads in shCTL and shTUT4/7 cells, respectively. **(d)** The poly(A) tail length profiles of terminally uridylated E sgRNA from shCTL (black) and shTUT4/7 (red) infected cells are shown. Uridylated reads represent 6.3% and 14.6% of total reads in shCTL and shTUT4/7 cells, respectively. Dots indicate the frequency of poly(A) tail length for each sample, and the horizontal bars show the mean frequency of poly(A) tail length per condition (n=3 for all conditions).

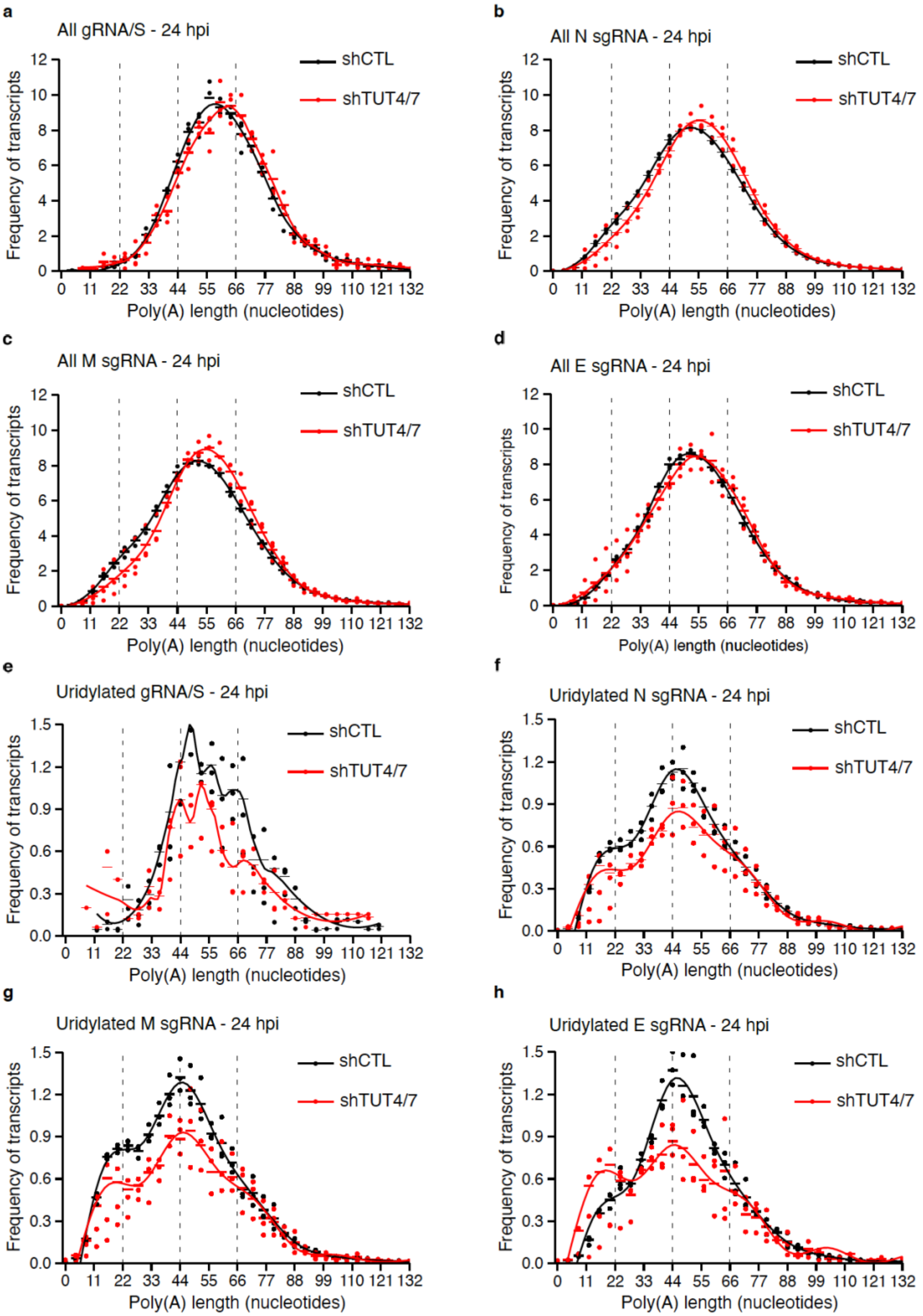
a
All gRNA/S - 24 hpi
b
All N sgRNA - 24 hpi
c
All M sgRNA - 24 hpi
d
All E sgRNA - 24 hpi
e
Uridylated gRNA/S - 24 hpi
f
Uridylated N sgRNA - 24 hpi
g
Uridylated M sgRNA - 24 hpi
h
Uridylated E sgRNA - 24 hpi
shCTL
shTUT4/7
Frequency of transcripts
Poly(A) length (nucleotides)
0 11 22 33 44 55 66 77 88 99 110 121 132
0 2 4 6 8 10 12
0.0 0.3 0.6 0.9 1.2 1.5

Supplementary Fig. 7. TUT4/7 do not affect the poly(A) profile of uridylated sgRNAs at 24 hpi. (a-d) The poly(A) tail profiles of the gRNA/S **(a)**, N sgRNA **(b)**, M sgRNA **(c)**, and E sgRNA **(d)** from shCTL (black) and shTUT4/7 (red) infected cells at 24 hours post-infection (hpi) are shown. **(e)** The poly(A) tail length profiles of terminally uridylated gRNA/S from shCTL (black) and shTUT4/7 (red) infected cells are shown. Uridylated reads represent 12.4% and 9.3% of total reads in shCTL and shTUT4/7 cells, respectively. **(f)** The poly(A) tail length profiles of terminally uridylated N sgRNA from shCTL (black) and shTUT4/7 (red) infected cells are shown. Uridylated reads represent 13.5% and 10.6% of total reads in shCTL and shTUT4/7 cells, respectively. **(g)** The poly(A) tail length profiles of terminally uridylated M sgRNA from shCTL (black) and shTUT4/7 (red) infected cells are shown. Uridylated reads represent 16.0% and 11.7% of total reads in shCTL and shTUT4/7 cells, respectively. **(h)** The poly(A) tail length profiles of terminally uridylated E sgRNA from shCTL (black) and shTUT4/7 (red) infected cells are shown. Uridylated reads represent 14.0% and 10.8% of total reads in shCTL and shTUT4/7 cells, respectively. Dots indicate the frequency of poly(A) tail length for each sample, and the horizontal bars show the mean frequency of poly(A) tail length per condition (n=3 for all conditions).
