# Supplementary material for: TUT4/7-mediated uridylation of a coronavirus subgenomic RNAs delays viral replication
Source: Commun Biol. 2023 Apr 21;6:438. doi: 10.1038/s42003-023-04814-1 (PMC10119532; doi:10.1038/s42003-023-04814-1)
Supplement: Supplementary file 2 — Description of Additional Supplementary Files [file 42003_2023_4814_MOESM2_ESM.pdf]

Description of Additional Supplementary Files

File name: Supplementary Data 1

Description: The source data behind Figure 3b
