# Supplementary material for: TUT4/7-mediated uridylation of a coronavirus subgenomic RNAs delays viral replication
Source: Commun Biol. 2023 Apr 21;6:438. doi: 10.1038/s42003-023-04814-1 (PMC10119532; doi:10.1038/s42003-023-04814-1)
Supplement: Supplementary file 4 — Reporting Summary [file 42003_2023_4814_MOESM4_ESM.pdf]

nature portfolio

Corresponding author(s): Marcos Morgan

Last updated by author(s): Mar 21, 2023

Reporting Summary

Nature Portfolio wishes to improve the reproducibility of the work that we publish. This form provides structure for consistency and transparency in reporting. For further information on Nature Portfolio policies, see our Editorial Policies and the Editorial Policy Checklist.

Statistics

For all statistical analyses, confirm that the following items are present in the figure legend, table legend, main text, or Methods section.

n/a	Confirmed	
☐	☒	The exact sample size (*n*) for each experimental group/condition, given as a discrete number and unit of measurement
☐	☒	A statement on whether measurements were taken from distinct samples or whether the same sample was measured repeatedly
☐	☒	The statistical test(s) used AND whether they are one- or two-sided<br>*Only common tests should be described solely by name; describe more complex techniques in the Methods section.*
☒	☐	A description of all covariates tested
☐	☒	A description of any assumptions or corrections, such as tests of normality and adjustment for multiple comparisons
☐	☒	A full description of the statistical parameters including central tendency (e.g. means) or other basic estimates (e.g. regression coefficient) AND variation (e.g. standard deviation) or associated estimates of uncertainty (e.g. confidence intervals)
☐	☒	For null hypothesis testing, the test statistic (e.g. *F*, *t*, *r*) with confidence intervals, effect sizes, degrees of freedom and *P* value noted<br>*Give P values as exact values whenever suitable.*
☒	☐	For Bayesian analysis, information on the choice of priors and Markov chain Monte Carlo settings
☒	☐	For hierarchical and complex designs, identification of the appropriate level for tests and full reporting of outcomes
☒	☐	Estimates of effect sizes (e.g. Cohen's *d*, Pearson's *r*), indicating how they were calculated

Our web collection on statistics for biologists contains articles on many of the points above.

Software and code

Policy information about availability of computer code

Data collection	Fasta5 files were generated by Nanopore MinKNOW software.<br>qPCR cycle threshold (Ct) values were obtained using the ABI PRISM 7900 Sequence Detection System and analysis software (Applied Biosystems).
Data analysis	Direct RNA-seq libraries were demultiplexed with DeePlexiCon and basecalled with Guppy version 5.0.10.<br>The demultiplexed reads were mapped using Minimap2 version 2.20.<br>The poly(A) tail length was determined using Nanopolish version 0.13.3.<br>Differential gene expression was determined using DESeq2.<br>The ontology of the differentially regulated genes was obtained using the TopGO R package.<br>Pathway analysis of differentially expressed was performed using Ingenuity Pathway Analysis (IPA).

For manuscripts utilizing custom algorithms or software that are central to the research but not yet described in published literature, software must be made available to editors and reviewers. We strongly encourage code deposition in a community repository (e.g. GitHub). See the Nature Portfolio guidelines for submitting code & software for further information.

Data

Policy information about availability of data

All manuscripts must include a data availability statement. This statement should provide the following information, where applicable:

- Accession codes, unique identifiers, or web links for publicly available datasets
- A description of any restrictions on data availability
- For clinical datasets or third party data, please ensure that the statement adheres to our policy

The direct RNA sequencing data has been deposited at the Gene Expression Omnibus (GEO) with accession number GSE200416

Human research participants

Policy information about studies involving human research participants and Sex and Gender in Research.

Reporting on sex and gender	*Use the terms sex (biological attribute) and gender (shaped by social and cultural circumstances) carefully in order to avoid confusing both terms. Indicate if findings apply to only one sex or gender; describe whether sex and gender were considered in study design whether sex and/or gender was determined based on self-reporting or assigned and methods used. Provide in the source data disaggregated sex and gender data where this information has been collected, and consent has been obtained for sharing of individual-level data; provide overall numbers in this Reporting Summary. Please state if this information has not been collected. Report sex- and gender-based analyses where performed, justify reasons for lack of sex- and gender-based analysis.*
Population characteristics	*Describe the covariate-relevant population characteristics of the human research participants (e.g. age, genotypic information, past and current diagnosis and treatment categories). If you filled out the behavioural & social sciences study design questions and have nothing to add here, write "See above."*
Recruitment	*Describe how participants were recruited. Outline any potential self-selection bias or other biases that may be present and how these are likely to impact results.*
Ethics oversight	*Identify the organization(s) that approved the study protocol.*

Note that full information on the approval of the study protocol must also be provided in the manuscript.

Field-specific reporting

Please select the one below that is the best fit for your research. If you are not sure, read the appropriate sections before making your selection.

☒ Life sciences ☐ Behavioural & social sciences ☐ Ecological, evolutionary & environmental sciences

For a reference copy of the document with all sections, see nature.com/documents/nr-reporting-summary-flat.pdf

Life sciences study design

All studies must disclose on these points even when the disclosure is negative.

Sample size	A sample size of n=3 was selected for all differential expression analyses, which is commonly accepted as the minimum number of samples for this type of analysis.
Data exclusions	No data were excluded from the analysis
Replication	All attempts at replication were successful
Randomization	This is not relevant to this study
Blinding	Sample handling was mostly performed by the same unblinded researcher throughout the experiments.

Reporting for specific materials, systems and methods

We require information from authors about some types of materials, experimental systems and methods used in many studies. Here, indicate whether each material, system or method listed is relevant to your study. If you are not sure if a list item applies to your research, read the appropriate section before selecting a response.

Materials & experimental systems

n/a	Involved in the study
☒	☐ Antibodies
☐	☒ Eukaryotic cell lines
☒	☐ Palaeontology and archaeology
☒	☐ Animals and other organisms
☒	☐ Clinical data
☒	☐ Dual use research of concern

Methods

n/a	Involved in the study
☒	☐ ChIP-seq
☒	☐ Flow cytometry
☒	☐ MRI-based neuroimaging

Eukaryotic cell lines

Policy information about cell lines and Sex and Gender in Research

Cell line source(s)	17-CL1 cell line, BEI Resources, Cat# NR-53719<br>NCTC clone 1469 cell line, ATCC, Cat# CCL-9.1
Authentication	None of the cell lines were authenticated
Mycoplasma contamination	Cell lines were tested for mycoplasma throughout the course of the experiments. Cells lines that tested negative for mycoplasma were propagated. Cell lines that tested positive were immediately discontinued and replaced with clean ones.
Commonly misidentified lines<br>(See ICLAC register)	N/A
